# Design of aptamer peptides for immunomodulation

**DOI:** 10.1080/14756366.2026.2717480

**Published:** 2026-09-02

**Authors:** Arpan Chowdhury, Prajesh Shrestha, Vivekanandan Subramanian, Venkatesh Mayandi, Mukesh Mahajan, Seetharama D. Jois

**Affiliations:** ^a^ Department of Pathobiological Sciences, School of Veterinary Medicine, Louisiana State University, Baton Rouge, LA, USA; ^b^ Department of Pharmaceutical Sciences, College of Pharmacy, University of Kentucky, Lexington, KY, USA; ^c^ Department of Chemistry and Biochemistry, University of California San Diego, San Diego, CA, USA

**Keywords:** Aptamer peptide, SFTI-1, CD2, CD58, immunomodulation

## Abstract

In this study, we describe the design of peptide aptamers to modulate interactions between CD2 and CD58 (co-stimulatory molecules) in the immune response. We designed peptide aptamers based on the sunflower trypsin inhibitor-1 (SFTI-1) template, incorporating functional groups that confer conformational stability and aqueous solubility. To address the challenges posed by conformational isomerism in the sunflower trypsin inhibitor template due to proline-proline sequences in peptide design, we developed two aptamer peptides, SFTI-FGUA and SFTI-DMY, incorporating specific functional groups. SFTI-FGUA features a side-chain guanidine group on phenylalanine, while SFTI-DMY features a dimethyl group at the meta position relative to tyrosine’s hydroxyl group. These bulky substitutions on the phenyl ring effectively restrict conformational flexibility and enhance solubility. Evaluation of these peptides using cell adhesion inhibition assays revealed that both aptamer peptides not only exhibited significant inhibition of cell adhesion but also exhibited a predominant conformation in solution. Furthermore, these peptides exhibited stability against enzymatic degradation.

The engagement of CD2 with CD58 in trans protein-protein interaction are crucial for the immune activation during T cell-antigen-presenting cell (APC) interactions. CD58 expression on antigen-presenting cells is involved in the initial stage of the immune response, and accumulation of these molecules at the intercellular cluster brings the two cells together, forming an immunological synapse with the help of other costimulatory molecules^1–3^. It is known that CD58 is overexpressed at the synovium of arthritis joints during the immune response of rheumatoid arthritis^4^. Knockout or knockdown of CD58 decreases the interaction of T cells and APCs by 100-fold, making CD58 an attractive target to modulate immune response. The interface of CD2 and CD58 interaction is dominated by salt bridges and hydrogen bonds. However, it is known that hydrophobic interactions act as hotspots in these two protein-protein interactions. A cation- π interaction and hydrophobic interaction of amino acids K, F, and Y hold the two proteins together, and upon mutation of these residues, that interaction weakens^5^. The nearly planar structure of proteins at the interface and the handshake interaction of β-sheets between two proteins, with hot-spot residues on the CD58 protein surface, make it a good target for peptides and peptidomimetics to modulate the immune response. Antibodies have been targeted to CD58 to disrupt the CD2-CD58 interaction, which can be used as therapy for inflammatory diseases^6^^,^^7^. Peptides and peptidomimetics for targeting CD58 and hence modulating the immune response have been reported in the literature^8–11^. Here, we describe aptamer peptides^12^^,^^13^ to modulate CD2-CD58 immune response.

Peptide aptamers are short chains made up of amino acids that can specifically bind to certain targets and have conformational constraints incorporated in the structure^12^. The design of aptamers is based on the target binding site, such as protein surfaces that are involved in protein-protein interactions. Aptamer design is based on the epitopes of protein partners or antibody-binding regions. Aptamer structures can be grafted onto template structures that are easily synthesised, chemically stable, resistant to enzymatic degradation, and water soluble^14^.

To render aptamers a level of structural stability similar to that of antibody binding sites, they are often designed as cyclic peptides. This design is thought to promote stronger binding compared to linear chains. We have designed peptide aptamers based on the template of sunflower trypsin inhibitor-1 (SFTI-1) and incorporated unnatural amino acids with soluble functional groups at strategic positions to render them conformational stability and aqueous solubility (Figures 1, 2, and S1). The designed peptides are named SFTI-phenyl guanidine (SFTI-FGUA) and SFTI-dimethyltyrosine (SFTI-DMY). The designed peptides were studied for cell adhesion inhibition assay, stability, and detailed three-dimensional structure. These peptide aptamers exhibit cell adhesion inhibition activity, a single major conformation in solution, aqueous solubility, and stability against gastric fluid, microsomal enzymes, and thermal degradation.

**Figure 1.**
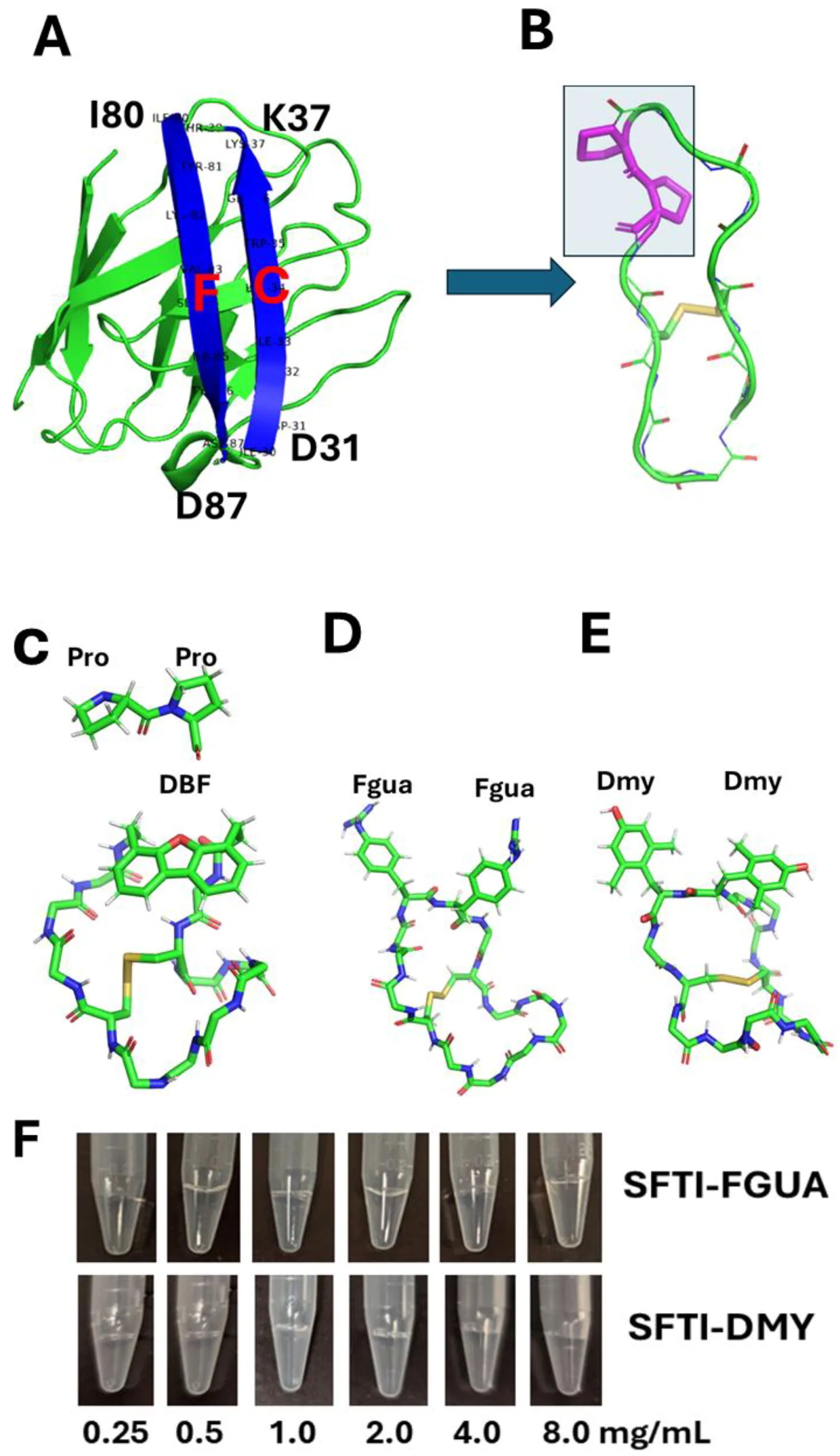
Design of aptamer peptides. (A) F and C strands of CD2 binding to CD58 (PDB ID 1QA9). (B) Amino acids F and C strands of CD2 were grafted onto the sunflower trypsin inhibitor template (PDB ID 1JBL). Proline-Proline sequence in the peptide is highlighted (boxed region). (C) Proline-proline sequence in the peptide was replaced with dibenzofuran moiety (DBF), resulting in aptamer peptide SFTI-DBF. DBF or proline-proline replaced with (D) phenyl guanidine – Fgua-Fgua or (E) Dimethyl tyrosine – Dmy-Dmy, resulting in aptamer peptides SFTI-FGUA and SFTI-DMY. 3D structure of DBF was generated using PyMol, Dahal et al^19^. and for FGUA and DMY, structures were generated using CYANA (L.A. Systems Inc.) and PyMol (Schrodinger Inc.). (F) solubility of SFTI-FGUA and DMY at different concentrations.

**Figure 2.**
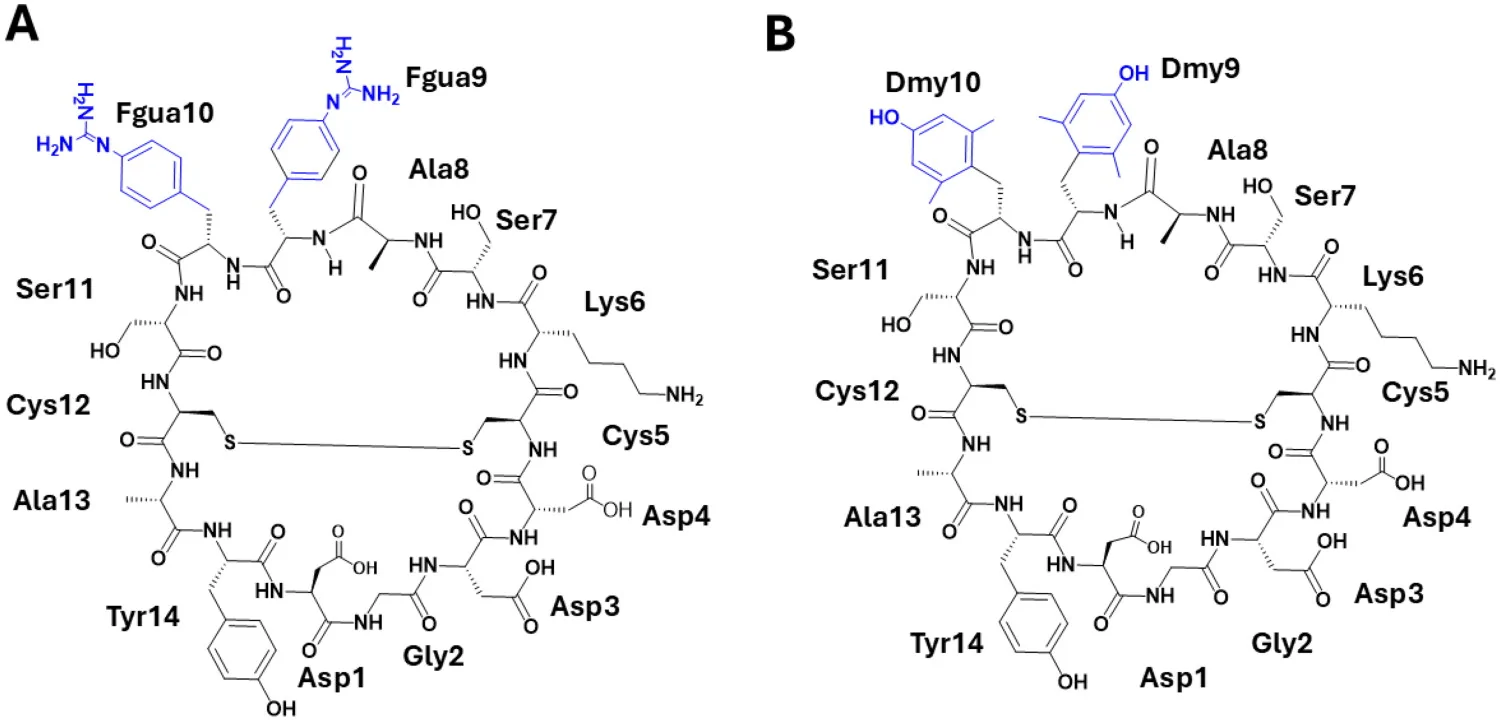
Chemical structures of (A) SFTI-FGUA and (B) SFTI-DMY showing amino acid residues and disulphide bond. Fgua and Dmy are shown in blue.

## Design of aptamer peptides

Structural studies have shown that the β-strands with charged residues of CD2 bind with CD58^5^^,^^7^ (Figure 1A), and peptides designed from amino acids sequences from β-strand region of CD2 inhibit the intercellular interaction between Jurkat cells and epithelial fibroblast cells^15^^,^^16^. These peptides also inhibited anti-CD2 antibody binding to CD58 on Caco-2 cells, suggesting their interaction with the CD58 protein adhesion domain^8^. Cyclic peptides such as SFTI-1 and rhesus θ defensin-1 possess disulphide-stabilised frameworks, making them highly resistant to proteolytic degradation^17^^,^^18^. To improve the stability of the CD2 epitope peptides, we used the aptamer method to generate stable peptides using the sunflower trypsin inhibitor-1 (SFTI-1) template (Figure 1B). It is reported that SFTI-1 grafted peptides inhibit cell adhesion and attenuate the immune response of murine T cells^9^. It is also reported that SFTI-DBF, a grafted peptide (Figure 1C) binds to the adhesion domain of CD58^11^^,^^19^ with a K_d_ of 46 nM. Furthermore, using flow-cytometry analysis, competitive binding of SFTI-DBF to CD58 protein expressed on the OVCAR-3 cells (expressing CD58) in the presence of FITC-AbCD58 was observed, suggesting that it inhibits protein-protein Interaction (PPI) of CD2-CD58^11^^,^^19^. However, the aqueous solubility of the reported peptides was limited. To overcome the limitations of the reported peptides, new peptides with aromatic functional groups (Figure 1D,E, Table S1) and substituted charged/hydroxy groups were designed to constrain conformation and enhance aqueous solubility, respectively (Figure 1F).

## Materials and methods

### Chemicals

Diisopropylethylamine (DIEA), N-methylmorpholine (NMM) and Triisopropylsilane (TIPS) were purchased from Sigma-Aldrich (St. Louis, MO). Piperidine, trifluoroacetic acid (TFA), Fmoc-protected amino acids, 2-chlorotrityl chloride resin, 1,1-bis(dimethylamino)methylene-1H-1,2,3-triazolo[4,5-b]pyridinium 3-oxide hexafluorophosphate (HATU) and 2–(6-chloro-1H-benzotriazole-1-yl)-1,1,3,3-tetramethylammonium hexafluorophosphate (HCTU) were sourced from Advanced ChemTech (Louisville, KY). N,N-Dimethylformamide (DMF) was obtained from Protein Technologies (Tucson, AZ). Additionally, Methylene chloride (DCM), methanol (MeOH), and acetonitrile (CH_3_CN) were procured from VWR International, LLC (Radnor, PA). Fmoc-L-(2,6-di-Me)Tyr-OH **(**DMY) and Fmoc-Phe(4-guanidino-Boc2)-OH **(**FGUA) were obtained from AnaSpec (Fremont, CA).

### Cell lines

Human epithelial ovarian adenocarcinoma cells (OVCAR-3) and human T-lymphocyte cells (Jurkat, E6-1) were procured from the American Type Culture Collection (ATCC). Additionally, human fibroblast-like synoviocytes derived from rheumatoid arthritis patients (HFLS-RA) were obtained from Cell Applications, Inc (San Diego, CA, USA), and cultured in a human synoviocytes growth medium supplied by the same company. Jurkat cells were maintained in RPMI-1640 medium (ATCC) supplemented with 10% foetal bovine serum (FBS), 0.1 mg/mL penicillin/streptomycin and 0.01 mg/mL bovine insulin. OVCAR-3 cells were also cultured in RPMI-1640 medium from ATCC but with 20% FBS, 0.1 mg/mL penicillin/streptomycin and 0.01 mg/mL bovine insulin. All cell lines were maintained in 5% CO_2_ at 37 °C in a humid atmosphere.

### Synthesis of peptide

Peptides were synthesised using solid-phase peptide synthesis (SPSS) as described previously by others^9^^,^^19^^,^^20^. The linear peptides, namely YDGDDCKSA-Fgua-Fgua-SCA-COOH (or YDGDDCKSA-DMY-DMY-SCA-COOH) were synthesised on a Chorus peptide synthesiser (Gyros Protein Technologies, Tucson, AZ) utilising a standard Fmoc peptide chemistry protocol on a 100 μmol scale using H-Ala-CTC resin (196 mg, 0.51 mmol/g). Five-fold excess of Fmoc-amino acids (Fmoc-Asp(OtBu)−OH, Fmoc-Cys(Thp)−OH, Fmoc-Lys(Boc)-OH, Fmoc-Ser(tBu)-OH, Fmoc-Ala − OH, Fmoc-Tyr(tBu)−OH, Fmoc-Phe(4-guanidine-Boc)-OH, and Fmoc-Asp(OtBu)-(Dmb)Gly-OH) and Oxyma Pure (Advanced ChemTech, Louisville, KY), in the presence of 5 equivalents of N,N-Diisopropylcarbodiimide (DIC) (Advanced ChemTech, Louisville, KY) were used for each of the amino acid coupling steps with N-Methyl-2-pyrrolidone (NMP) (Advanced ChemTech, Louisville, KY) as the solvent at 75 °C. After synthesis, the resin was washed with N,N-Dimethylformamide (DMF) and Dichloromethane (DCM) (Advanced ChemTech, Louisville, KY). The linear peptide was cleaved from the resin with the aid of 1% Trifluoroacetic acid (TFA) (Advanced ChemTech, Louisville, KY) in DCM. After the solvent was vacuumed, the residue was dissolved in 50 ml DMF, 95 mg 2–(7-Aza-1H-benzotriazole-1-yl)-1,1,3,3 – tetramethylaminium hexafluorophosphate (HATU) (Advanced ChemTech, Louisville, KY) and 87 μL of DIEA and stirred for 2 h at room temperature, followed by the removal of solvent by a rotary evaporator. A mixture of TFA: water: TIPS (4 ml, 95:2.5:2.5) was added to the residue and stirred for 2 h. Then, cold diethyl ether was added to the peptide solution to precipitate, and the mixture was centrifuged for 10 min (4000 rpm). Fresh cold diethyl ether was added, and the pelleted peptide was resuspended, frozen, and lyophilised. The crude cyclized peptide was dissolved in 50% acetonitrile/water (0.1% TFA) at a concentration of 2 mM. The iodine saturated in 50% acetonitrile/water solution (containing 0.1% TFA) was slowly added to this solution under stirring until the solution turned yellow. After 1 h of stirring, the reaction was quenched using a saturated solution of ascorbic acid until the peptide solution turned colourless, followed by lyophilisation to obtain the final peptide. A peptide conjugate of 6-carboxyfluorescein (6-FAM) was synthesised by attaching the fluorescent molecule 6-FAM to the peptide via the amino acid Lys side chain.

Peptide analysis and purification were carried out using the Thermo Scientific Dionex Vanquish system. Chromeleon 7.3.1 software was used to control the system and process data. The sample was separated on a Waters XSelect CSH C18 column (5 µm, 4.6 × 250 mm) with an XSelect CSH C18 guard column (5 µm, 4.6 × 20 mm) at room temperature. The mobile phase consisted of eluent A (0.1% TFA in water) and eluent B (0.1% TFA in acetonitrile). Gradient method (5%-55% of B/50 min) was used at a flow rate of 1.0 ml/min. The detected wavelength was set at 215 nm. The injected sample concentration and volume were 0.5 mg/ml and 20 μL, respectively.

### Solubility studies

Peptides SFTI-FGUA and DMY were dissolved in water with increasing concentration of the peptide (0.025 to 8 mg/mL) (United States Pharmacopoeia 2021 General Chapter, 1503, Quality Attributes of Synthetic Peptide Drug Substances. USP-NF), and the tube containing the peptide was stirred and kept on a rack for at least five minutes. The solution was tested for a homogeneous solution that is clear without any precipitation^21^.

### E-rosetting assay

Sheep red blood cells (SRBCs) (Colorado Serum Company, Denver, CO) were isolated by centrifuging sheep blood in Phosphate Buffered Solution (PBS) (Teknova, Hollister, CA) at 1000 *g* for 10 min followed by PBS washing (thrice), centrifugation at 1000 g for 10 min, and incubated with 4 volumes of 2–(2-aminoethyl)isothiourea dihydrobromide (AET) (Sigma Aldrich, St Louis, MO) solution at 37 °C for 20 min. Thereafter, PBS wash was given thrice and resuspended in RPMI-1640 (ATCC, Manassas, Virginia) containing 10% FBS to achieve a final SRBC concentration of 4% (v/v). Serial dilutions of aptamer peptides (SFTI-FGUA & SFTI-DMY) in PBS were added to 100 µL of 0.5% AET-treated SRBCs and incubated at 37 °C for 30 min. Subsequently, 100 µl of the Jurkat T cell suspension (2.5*10^6^/mL) was added to the mixture, which was gently rolled and incubated for an additional 15 min. Cells were pelleted by centrifugation (400 *g*, 5 min, 4 °C) and then incubated at 4 °C for 1 h. The cell pellet was gently resuspended, and the E-rosettes were enumerated using bright field microscopy. Rosettes were counted when Jurkat T cells were bound with five or more SRBCs. To determine the percentage of E-rosette cells at least 200 Jurkat T cells were counted. Following equation was used to calculate the inhibitory activity^22^^,^^23^:

Inhibition (%) = ((A − B)/C) * 100

A = % of non-E-rosette-forming cells treated with the compound or antibody.

B = % of non-E-rosette-forming cells without the compound.

C = % of E-rosettes formed in non-treated cells.

Experiments were repeated and IC_50_ was reported as mean ± standard deviation (S.D) of three IC_50_ values calculated.

### Competitive binding assay with fluorescently labelled peptide

OVCAR-3 cells were used for the competitive binding experiment. 10,000 cells/well were seeded in a 96-well tissue culture clear-bottom black plate and incubated overnight at 37 °C with 5% CO_2_ for the cells to attach to the plate. The next day, the medium was discarded, and the 96-well plate was washed thrice with PBS, followed by treatment with different concentrations of SFTI-DMY and SFTI-FGUA (0.78-100μM) for 1 h and later with SFTI-FAM-FGUA conjugate (25 μM) at 37 °C with 5% CO_2_ to allow the aptamers and SFTI-FAM-FGUA conjugate to compete. Subsequently, the sample was washed thrice with PBS, followed by fluorescence intensity measurement with a microplate reader at 495 nm and 525 nm as the excitation and emission wavelengths, respectively. Concentration vs. relative fluorescence from the average fluorescence intensity was used to plot the graph^24^. Results are expressed as mean ± S.D

### Cell viability studies in the presence of aptamer peptides

Cell viability assay was performed on OVCAR-3, HFLS-RA and Jurkat T cells (ATCC, Manassas, Virginia) using CellTiterGlo (Promega, Madison, WI)^25^. 10,000 cells/well were seeded and incubated for 24 h. Subsequently, aptamer peptides were added and incubated for 24 h with concentrations ranging from 0.1 μM to 50 μM. Aptamer peptides were discarded and CellTiterGlo was added and incubated for 20 min, followed by luminescence reading using plate reader (BioTek, Winooski, Vermont). Cells without the aptamer peptides and with 1% DMSO were used as negative and positive controls, respectively. The viability of the OVCAR-3, HFLS-RA, and Jurkat T cells was calculated relative to cells without the aptamer peptides. Results were represented from triplicate values (mean ± S.D).

### Circular dichroism (CD) spectroscopy

JASCO-J800 spectrophotometer was used to acquire the CD spectra. Peptides were dissolved in water: methanol (1:1) to obtain a final concentration of 50 μM. CD scans were performed over a wavelength range of 190 nm to 300 nm, and 10 scans were averaged to produce the final spectra. Baseline blank was subtracted from the peptide spectra. To assess the stability of the aptamer peptides at varying temperatures, the CD cuvettes were placed in a block connected to a water-circulation system with a heater. The temperature of the spectrophotometer slowly increased from 25 °C to 65 °C to acquire CD spectra at a particular temperature with ±0.5 deg. The average CD spectra was analysed qualitatively and quantitatively by BestSel tool for decomposition of CD spectra into different components^26^^,^^27^.

### Surface plasmon resonance (SPR) analysis

SPR analysis was performed using Biacore 1K (GE Health Care Life Sciences, Pittsburgh, PA). CD58 was purchased from Abcam (Cambridge, UK) and was immobilised in flow cell 4 on a CM5 SPR sensor chip (GE Healthcare Life Sciences) at 15 *µ*g/mL in pH 4.5 acetate buffer at a flow rate of 5 *µ*L/min, keeping flow cell 3 as a reference. Various concentrations of SFTI-FGUA and SFTI-DMY (1.5625 μM −200 μM) prepared in 1.02× PBS-P + buffer (GE Healthcare Life Sciences) were passed through at a flow rate of 20 μL with 120s of association time and 180s of dissociation time for the recording of binding response. When the ligand (SFTI-FGUA/DMY) binds with the immobilised CD58 protein, there is a change in the refractive index, which gives the binding response. Biacore 1K software was used to determine the K_d_ value by fitting the observed binding response curve using the 1:1 Langmuir equation.

### NMR spectroscopy

A total of 1.5 mg of SFTI-FGUA or DMY was dissolved in a solvent mixture of 90% H_2_O and 10% D_2_O. NMR experiments were recorded at 298 K on a Bruker Avance NEO 600 MHz spectrometer. Two-dimensional NMR experiments included DQF-COSY^28^, TOCSY (using an MLEV-17 spinlock with an 80-ms mixing time) ^29^, ROESY, and NOESY (300-ms mixing time)^30–32^. A relaxation delay of 1.5 s was used for all 2D NMR experiments.

The 2D NOESY and TOCSY datasets were acquired with 2048 data points in the direct dimension (t_2_) and 512 increments in the indirect dimension (t_1_) using the States-TPPI method. For DQF-COSY, datasets consisted of 4096 data points in t_2_ and 512 increments in t_1_, also acquired using the States-TPPI method. Prior to complex Fourier transformation, free induction decays (FIDs) were multiplied by squared, phase-shifted sine-bell window functions. The 2D datasets were zero-filled to 2048 real points in both dimensions, resulting in a digital resolution of 2.23 Hz/point. Spectral processing was performed using Bruker TopSpin 4.1.4, and water resonance at 4.77 ppm was used as a reference to assign different chemical shifts.

Vicinal^3^JCαH–NH coupling constants were determined from 1D ^1^H NMR spectra. All 2D NMR data were analysed using SPARKY 3.190^33^, and peak lists were exported for structure calculations using CYANA version 2.1^34^. Temperature-dependent 1D ^1^H and 2D TOCSY spectra facilitated identification of changes in amide proton chemical shifts within the peptide^35^.

Additionally,^13^C–^1^H HSQC and ^15^N–^1^H HSQC spectra^36^ were recorded on the same 600 MHz spectrometer equipped with a triple-resonance TXI cryoprobe. The 1H spectral width was set to 9090.90 Hz, and water suppression was achieved using the excitation sculpting method. The indirect dimensions of both HSQC experiments were acquired with 2K × 128 points.

### Structure calculations using CYANA

Assigned NOESY peaks were categorised as weak, medium, or strong and translated into upper bound distance restraints of 5.0 Å, 3.5 Å, and 2.8 Å, respectively. The structures of peptides incorporating the non-canonical amino acids FGU and DMY were calculated using CYANA 2.1^37^. Custom CYANA library entries for FGU and DMY were manually created (libraries with atom names are provided at the end of the supporting information). Briefly, in PyMOL, the side chains of the standard amino acids phenylalanine (Phe) and tyrosine (Tyr) were manually modified to generate the non-canonical residues FGU and DMY, respectively. The coordinate values of these modified residues were incorporated into the CYANA library for structure calculations. During the CYANA runs, residues ASP-1 and TYR-14 were covalently linked via an amide bond, and a disulphide bond was introduced between the thiol groups of CYS-4 and CYS-12. Structure calculations were carried out in a stepwise manner to obtain a final ensemble with low root-mean-square deviation (RMSD) and minimal violations of distance and dihedral angle restraints.

### MD simulations

The simulation of the peptide was performed using NAMD 2.14 on the High Performance Cluster (HPC). The structure of the peptide was prepared within the Molecular Operating Environment (MOE 2024.0601; Chemical Computing Group, Montreal, Canada). Initially, geometry cleanup and protonation were performed at physiological pH 7.0. Parameters and charges were assigned using the Amber10: EHT forcefield. Particle Mesh Ewald (PME) method was used to calculate long-range electrostatic interactions and Lennard-Jones interactions were treated with a switching scheme from 8 to 10 Å.

The system was solvated with water of approximately 1.001 g/cm^3^ in a periodic orthorhombic water box of dimension 45.5 × 40.0 × 40.0 Å³. For the SFTI-DMY and SFTI-FGUA peptide, the amide bond between Tyr 14 and Asp1 was tethered to maintain the dihedral angle during equilibration and production. The system was equilibrated through a multistep protocol.

Initially, a 10 picosecond (ps) energy minimisation was performed to remove clashes and relax the system, followed by a 100 ps heating phase, gradually increasing the temperature from 100 K to 300 K under constant volume (NVT) conditions. Subsequently, a 100 ps hold time was applied at 300K to stabilise the system. Finally, pressure equilibration for 200 ps under the NPT ensemble at 300K and 100 kilopascals (kPa) was done to equilibrate system density and box volume. After equilibration, a 100 nanosecond (ns) production run was carried out in NPT ensemble at 300K and 100 kPa. Trajectories were saved at every 10 ps checkpoint^38^.

System stability was evaluated by monitoring the temperature, pressure, and total energy throughout the equilibration and production. Post-simulation trajectory analysis was done. The trajectories were analysed for RMSD relative to the initial coordinates. Similarly, the dihedral angle for Cβ-S-S-Cβ and the distance between Cα-Cα of the Cysteine residues were calculated.

### Docking

Docking was performed in Molecular Operating Environment (MOE 2024.0601; Chemical Computing Group, Montreal, Canada). The crystal structure of the adhesion complex between CD2 and CD58 (PDB ID:1QA9) was downloaded from the Protein Data Bank (PDB). CD58 (Chain D: residue 1–98) was used as a receptor. Using structure preparation, hydrogen atoms were added, and protonation states at pH 7.4 were assigned using Protonate3D. The entire chain D was defined as a binding site. For the ligands, 3D structures and multiple low-energy conformational states of the aptamers SFTI-FGUA and SFTI-DMY were generated in MOE and subsequently docked into the prepared receptor. Ligand placement was carried out using the Triangle matcher algorithm and initially scored using London dG, followed by rigid receptor refinement and rescoring using GBVI/WSA Dg force field-based scoring function. Finally, docking scores for aptamer peptides were recorded, and binding interactions were evaluated.

### Thermal stability

SFTI-FGUA and SFTI-DMY were dissolved in milliQ water at a concentration of 200 µM (400 µl), and Circular Dichroism was used to determine the secondary structure perturbation at 25 °C and 65 °C. The “Temperature Interval Measurement” mode of JASCO 815 (Jasco Inc., Easton, MD) CD spectrometer was used to measure the ellipticity of the peptides as a function of temperature. Ten scans were taken for each temperature, and the resulting graphs were plotted against the wavelength range 190 nm to 320 nm. HPLC and MALDI-TOF MS were performed on the peptides incubated at 70 °C at 1 mM concentration at different time points (0, 3, 6, 12, 24 & 48 h) to analyse their thermal stability.

### Chemical stability

To ensure the integrity of the disulphide bond in SFTI-FGUA and SFTI-DMY (200 µM), CD spectra were recorded in the presence of a reducing agent, i.e. Dithiothreitol (DTT), at concentrations of 0.1, 1, and 5 mM, and incubated for 30 minutes^9^. The samples were lyophilised after the CD spectrometry experiment and then analysed by MALDI-TOF MS.

### Stability of the peptides in simulated gastric fluid (SGF)

SGF and Pepsin were procured from Ricca (Cat No – 7108–32) and Sigma (Cat No – P700), respectively. SFTI-FGUA and SFTI-DMY were dissolved in SGF at a concentration of 1.6 mg/1.6 ml along with pepsin at a concentration of 3.2 mg/mL and incubated at 37 °C/100 rpm shaking. The enzyme: peptide ratio was maintained at 1:50. At different time points (0,15min, 30 min, 1, 2, 4, 6 & 24 h), 180 µL of the mixture was aspirated and added to 720 µL of chilled methanol, followed by centrifugation at 10,000 rpm/10 minutes^39^. The supernatant was aspirated, lyophilised, and subjected to HPLC. A gradient of 5%-45% solvent B was used for 50 min in the HPLC. Solvent A – 0.1% TFA/water and Solvent B – 0.1% TFA/acetonitrile. Somatostatin was used as a positive control.

### Stability of the peptides in simulated intestinal fluid (SIF)

SIF and Pancreatin (porcine) were procured from Ricca (Cat No – 7109.75–16) and Sigma (Cat No – P7545), respectively. SFTI-FGUA and SFTI-DMY were dissolved in SIF (pH-6.8) at a concentration of 1.7 mg/1.7 ml, followed by the addition of pancreatin (final concentration – 1%) and incubated at 37 °C/100 rpm shaking. At different time intervals (0, 15, 30 min, 1, 2, 4 6, & 24 h), 212.5 µL was aspirated and neutralised with 850 µL of 0.1 N HCl, followed by centrifugation at 10,000rpm/10 min The supernatant was aspirated, lyophilised and subsequently HPLC was performed on the reconstituted peptide by following a gradient method (5%-45% solvent B for 50 min). The compositions of Solvent A & B were 0.1% TFA/water and 0.1% TFA/acetonitrile respectively.

### Stability of the peptides in human liver microsomes

Human pooled liver microsome, NADPH, and CaCl_2_ were procured from Gibco (Cat No – HMS9PL), Millipore (Cat No – 10107824001), and Sigma (Cat No – C5670), respectively. 200 µL of 40 mM CaCl_2_ was added to 200 µL of 10 mM NADPH, 50 µL of human pooled liver microsome (20 mg/mL), 100 µL of 1 mM SFTI-FGUA (same was repeated for SFTI-DMY) and the volume was made up to 2000 µL by adding 1450 µL of milliQ water, which made 4 mM of CaCl_2_, 1 mM NADPH, 0.5 mg/mL human pooled liver microsome and 50 µM peptide as the final concentration^40^^,^^41^. Verapamil was used as a positive control. The mixture was incubated at 37 °C with shaking at 100 rpm. At discrete time points (15 and 30 min, 1, 2, 4, 6, and 24 h), 250 µL of the mixture was pipetted into 750 µL of chilled acetonitrile, chilled on ice for 2 min, followed by centrifugation at 10,000 rpm for 10 min. The supernatant was passed through a 0.2 µm filter, and then lyophilised. The HPLC was performed using a gradient method: 5%-45% solvent B over 50 min. The compositions of Solvent A & B were 0.1% TFA/water and 0.1% TFA/acetonitrile respectively.

Stability data from SGF and human liver microsomes were analysed by DDSolver. The percentage of peptide degradation kinetics was fitted to zero, first and second order kinetic models in DDSolver^42^. The goodness of fit was assessed using adjusted R^2^ and mean squared error (MSE).

## Results

### Synthesis and characterisation

Peptides SFTI-FGUA and SFTI-DMY were synthesised using solid-phase peptide synthesis as described in the literature^20^^,^^43^. Conjugate of SFTI-FGUA-6-FAM was synthesised as described in the literature^44^. Peptides were >90% pure, major HPLC peak and MALDI-TOF mass spectroscopy showed the molecular ion for the peptides with m/z 1623.078, 1597.078 and 1980.671 respectively, for SFTI-FGUA, SFTI-DMY, and SFTI-FGUA-6-FAM (Figures S2–S8).

### E-rosetting assay

**Figure 3.**
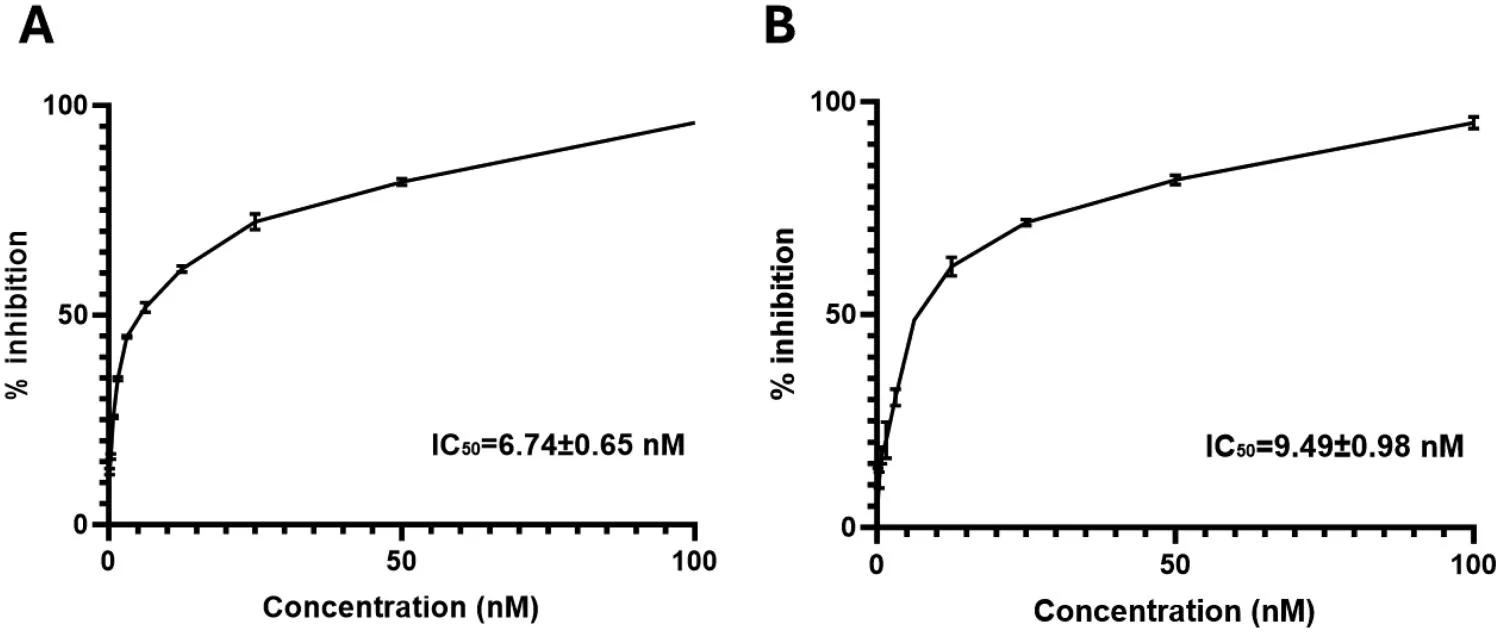
Evaluation of peptide cell adhesion inhibition activity by the E-rosetting assay. Dose response curves for A) SFTI-FGUA and B) SFTI-DMY. IC_50_ values were calculated using GraphPad Prism. Data represented as mean ± SD

The E-rosetting assay is a well-established model system for evaluating cell adhesion via the CD2-CD58 interaction^22^^,^^23^. The assay was performed in absence of the two aptamer peptides and at different concentrations of these aptamer peptides, and resulting e-rosetting formation was evaluated. Dose response curves (Figure 3) indicated that FGUA exhibited inhibition of e-rosetting with an IC_50_ value of 6.74 ± 0.65 nM and DMY with 9.49 ± 0.98 nM, respectively. An antibody with known binding affinity for CD58 was used as control. Representative images of positive and negative e-rosetting assay are provided in Figures S9–S11.

### Competitive binding assay

**Figure 4.**
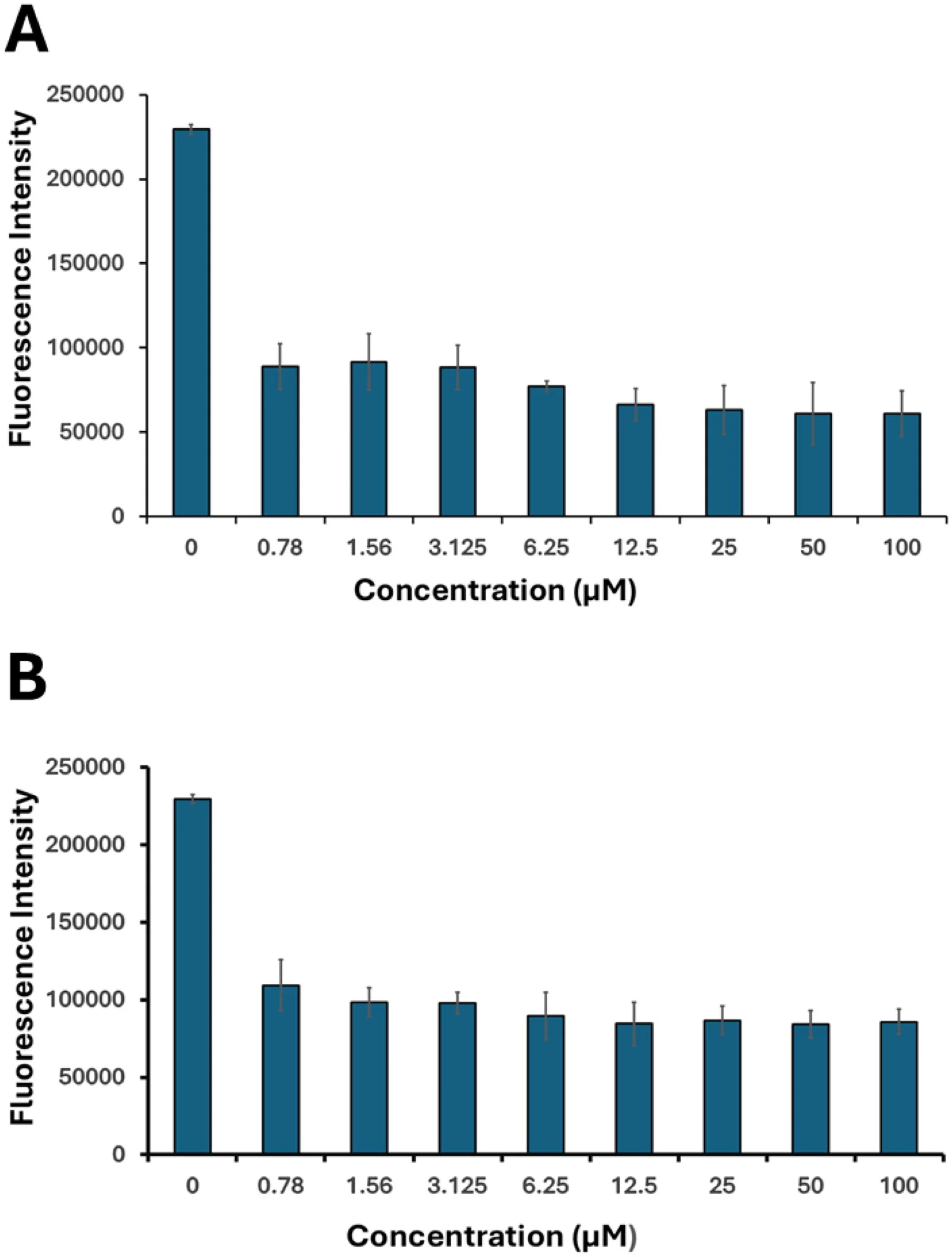
Competitive binding of peptides with fluorescently labelled SFTI-FGUA-6FAM on CD58-expressing OVCAR-3 cells monitored by fluorescence. (A) Concentration of SFTI-FGUA was varied at a constant concentration of SFTI-FGUA-6-FAM (25 μM). As unlabelled peptides bind to CD58 on cells, the fluorescently labelled peptide is displaced, decreasing the fluorescence intensity. (B) Binding of SFTI-DMY in the presence of SFTI-FGUA-6-FAM. Results are plotted as mean ± SD

OVCAR-3 cells are known to express CD58 protein and used as model for studying CD2-CD58 interactions^9^^,^^45^. Expression of CD58 on OVCAR-3 cells was confirmed by flow-cytometry using phycoerythrin (PE)-antibody to CD58 (Figure S12). As an orthogonal assay to the E-rosetting and surface plasmon resonance assays, a competitive binding assay was performed. We used the binding of fluorescently labelled peptide to CD58-expressing OVCAR-3 cells to detect the binding of unlabelled peptides SFTI-FGUA and DMY to OVCAR-3 cells. When unlabelled peptide SFTI-FGUA was added at different concentrations, the fluorescence intensity of SFTI-FGUA-6FAM decreased, suggesting replacement of unlabelled peptides. The binding of SFTI-FGUA was observed at less than 1 µM concentration. Similar results were observed for SFTI-DMY (Figure 4A,B)

### Cell viability studies in the presence of aptamer peptides

SFTI-FGUA and SFTI-DMY inhibit cell-cell adhesion between Jurkat and SRBC. We wanted to investigate whether cell adhesion inhibition results from the peptide’s toxicity. We incubated the peptide with Jurkat T cells (non-adherent cells) for 6 h, ovarian cancer cells (OVCAR-3 cells) for 24 h, and human fibroblast-like synoviocytes derived from rheumatoid arthritis (HFLS RA cells) for 24 h. Both these cells express the CD58 protein. The peptide concentration used was from 0.1 to 50 µM. Neither peptide showed significant toxicity in the cell lines used during incubation, suggesting that the cell adhesion inhibition was likely due to PPI inhibition rather than toxicity. As control, 0.05% SDS and 1% DMSO were used (Figures S13–S15).

### Circular dichroism (CD) spectroscopy

**Figure 5.**
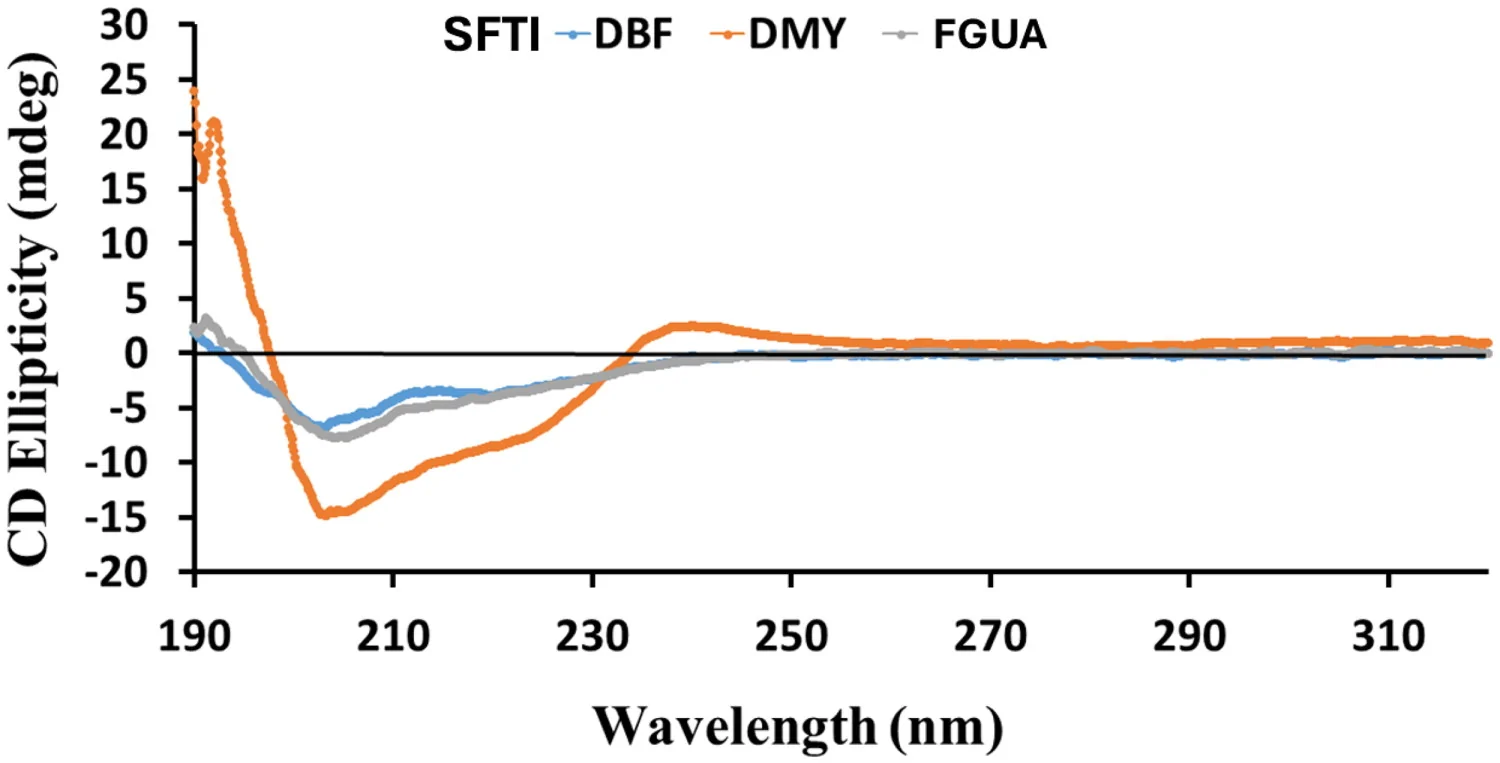
CD spectra of SFTI-FGUA and DMY. For comparison purposes, CD spectrum of the DBF peptide is also provided. Concentration of the peptide 50 µM, path length 1 mm. Spectra are the average of 10 scans.

CD spectra of SFTI-FGUA exhibited a negative band around 205 nm and a positive band around 190 nm. SFTI-DMY exhibited negative bands, around 205 and a negative shoulder around 225 nm, and a positive band around 190 nm (Figure 5). Perczel and Fasman have described different types of beta turns in peptides by deconvolution of CD spectra. A negative band around 205 nm and a positive band around 195 nm as well as negative band around 220 to 230 nm and positive band around 195 nm indicate two forms of type I β-turns. Thus both FGUA and DMY exhibit type 1 β-turn^46^. For comparison, SFTI-DBF, another peptide reported^11^, is represented. SFTI-DBF was shown to acquire a β-turn structure. However, the intensity of the negative band around 205 nm in SFTI-DMY was stronger compared to SFTI-DBF, suggesting that DMY may exhibit a twisted β-turn structure with the possibility of a small percentage of helical structure. Analysis of the CD spectra using BestSel suggested that SFTI-FGUA exhibits nearly 35% of β-turn/β-sheet structure, and SFTI-DMY exhibits 37% of β-turn/β-sheet structure. BestSel analysis also indicated that both peptides exhibit 14% and 19% right-twisted β-sheet structures [47] (Figures S16 and S17). Overall, CD studies suggest that both peptides exhibit β-turn structure.

### Structure of peptides

**Figure 6.**
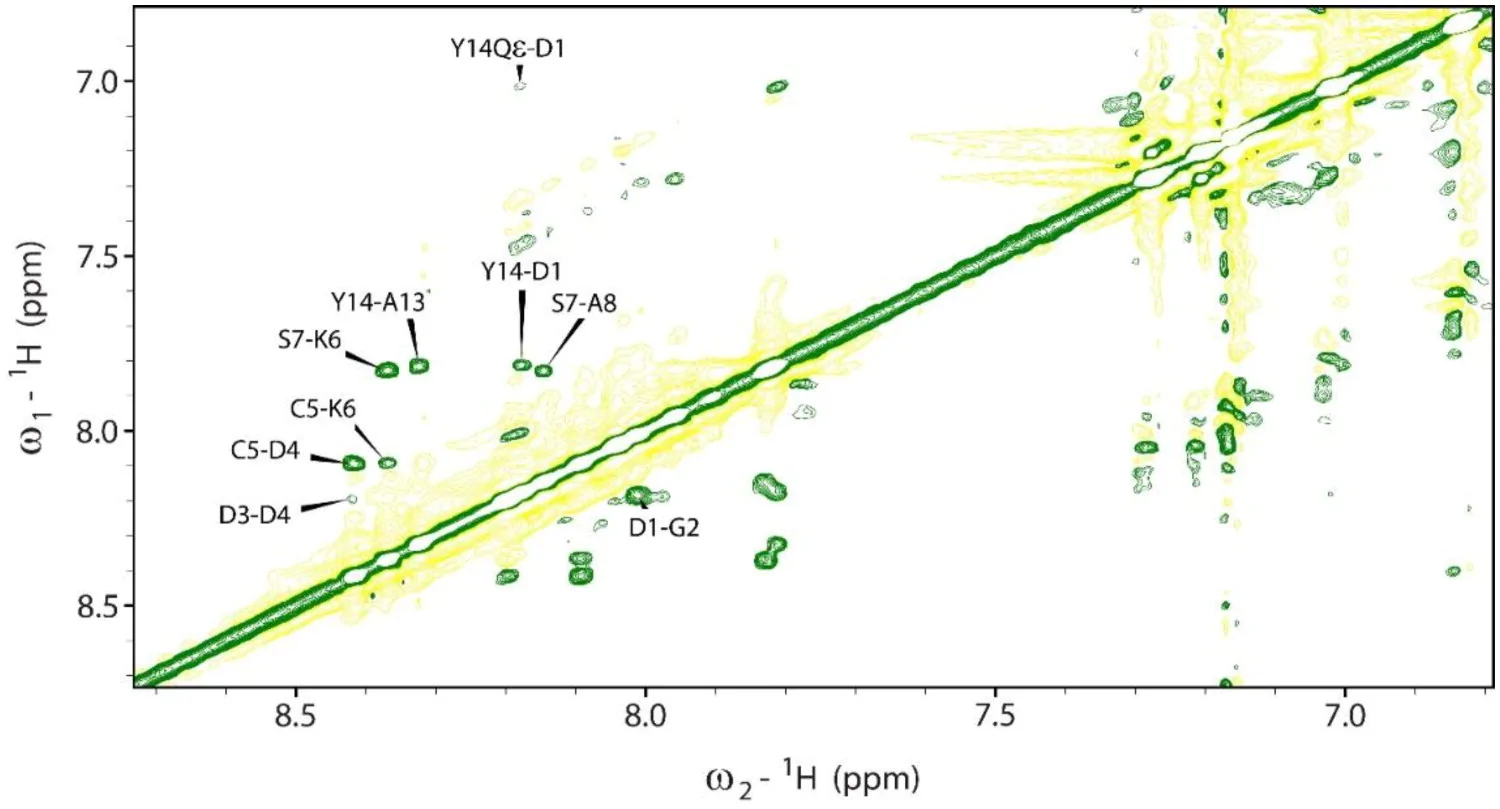
600 MHz ^1^H 2D NMR NOESY spectra for SFTI-FGUA. The NH-NH region of the NOESY spectra with assignments is shown. SFTI-FGUA was dissolved in a solvent mixture of 90% H_2_O and 10% D_2_O. For details, see the text.

**Figure 7.**
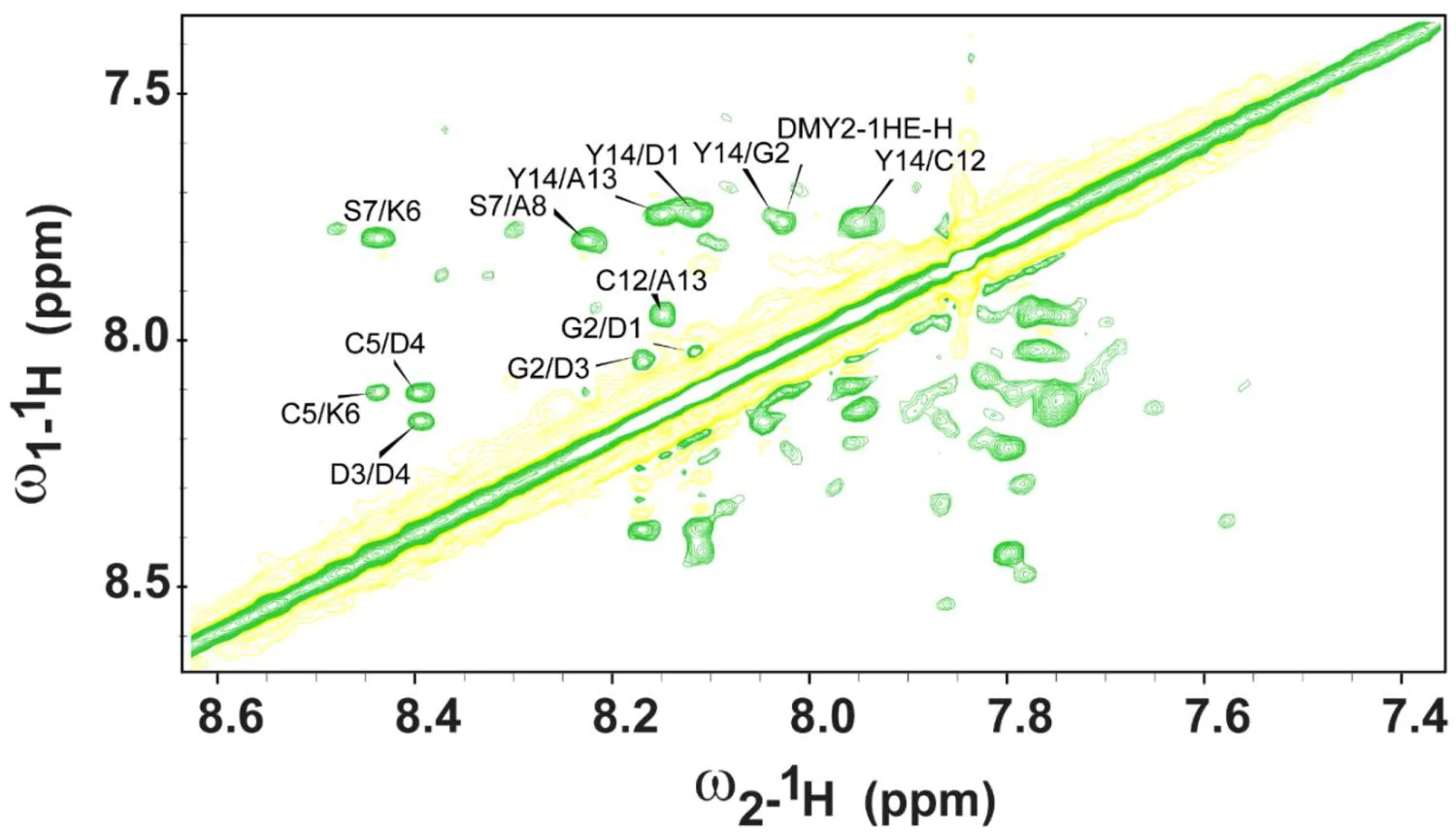
600 MHz ^1^H 2D NMR NOESY spectra for SFTI-DMY. The NH-NH region of the NOESY spectra with assignments is shown. SFTI-DMY was dissolved in a solvent mixture of 90% H_2_O and 10% D_2_O. For details, see the text.

**Figure 8.**
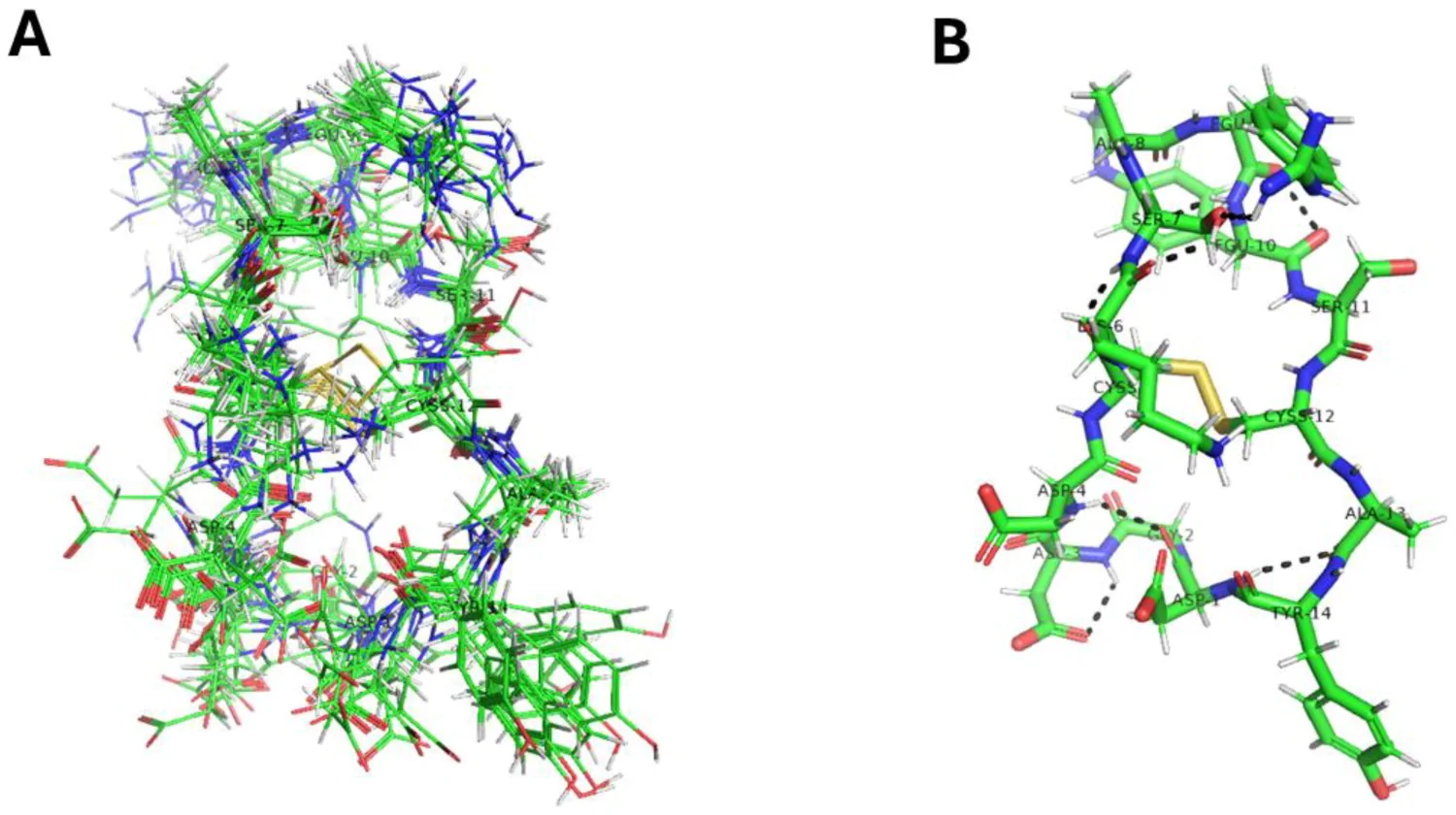
Ensemble of 10 NMR-derived structures using CYANA for (A) SFTI-FGUA and (B) one representative structure of SFTI-FGUA.

NMR spectra of SFTI-DMY and SFTI-FGUA were acquired in water. Each amino acid in the peptide showed only one major resonance, which indicates that the peptides exhibit one major conformation in solution. 1D and 2D NMR spectra were used to assign the resonances and spatial connectivity of protons^31^ in the peptide, which were obtained by NOESY spectra (Figures 6 and 7). Coupling constants were measured by DQF-COSY as well as well-resolved 1D NMR resonances of the amide peaks. 2D NMR TOCSY, DQF-COSY,^15^N, ^13^C HSQC, and NOESY spectra, along with chemical shift and coupling constants (Tables S2 and S3, Figures S18–S27). The NMR data were used to generate the peptide structures using CYANA, as described above (Tables 1 and 2). The structure of SFTI-FGUA exhibited two β-turns and two γ-turns and a disulphide bond stabilising the structure. The β-turns were around D1-G2-D3-D4 and S7-A8-Fgua9-Fgua10 (Figure 8). The phi and psi values around G2 and D3 were −60^º^, −17^º^ and −95^º^, −50^º^. Phi, Psi values around A8 and Fgua9 were −50^º^, 50^º^ and −56^º^, −17^º^. These turns are classified at type I β-turns. The γ-turns were around A13-Y14-D1 and C5-K6-S7. Hydrogen bonding stabilised both these turns. The NHs of D1, D4, K6, and Fgua10 exhibited hydrogen bonding with backbone carbonyl carbons of the peptide. The low temperature coefficient of the amide chemical shifts supported the hydrogen bonding between D4 and K6. The disulphide bond had a dihedral angle of −100°.

**Table 1.** A summary of structural statistics for twenty low energy structures of FGUA.

Distance constraints: Intra-residue [i = j]	58
Sequential [| i – j | = 1]	45
Medium range [1 < | i – j | < 4]	06
Long range [| i – j | ≥ 4]	09
Total	118
Dihedral-angle constraints	10
Deviation from mean structure All backbone atoms	0.78 Å
All heavy atoms	1.47 Å

**Table 2.** A summary of structural statistics for ten low energy structures of DMY.

Distance constraints: Intra-residue [i = j]	60
Sequential [| i – j | = 1]	56
Medium range [1 < | i – j | < 4]	10
Long range [| i – j | ≥ 4]	13
Total	139
Dihedral-angle constraints	10
Deviation from mean structure All backbone atoms	0.04 Å
All heavy atoms	0.45 Å

**Figure 9.**
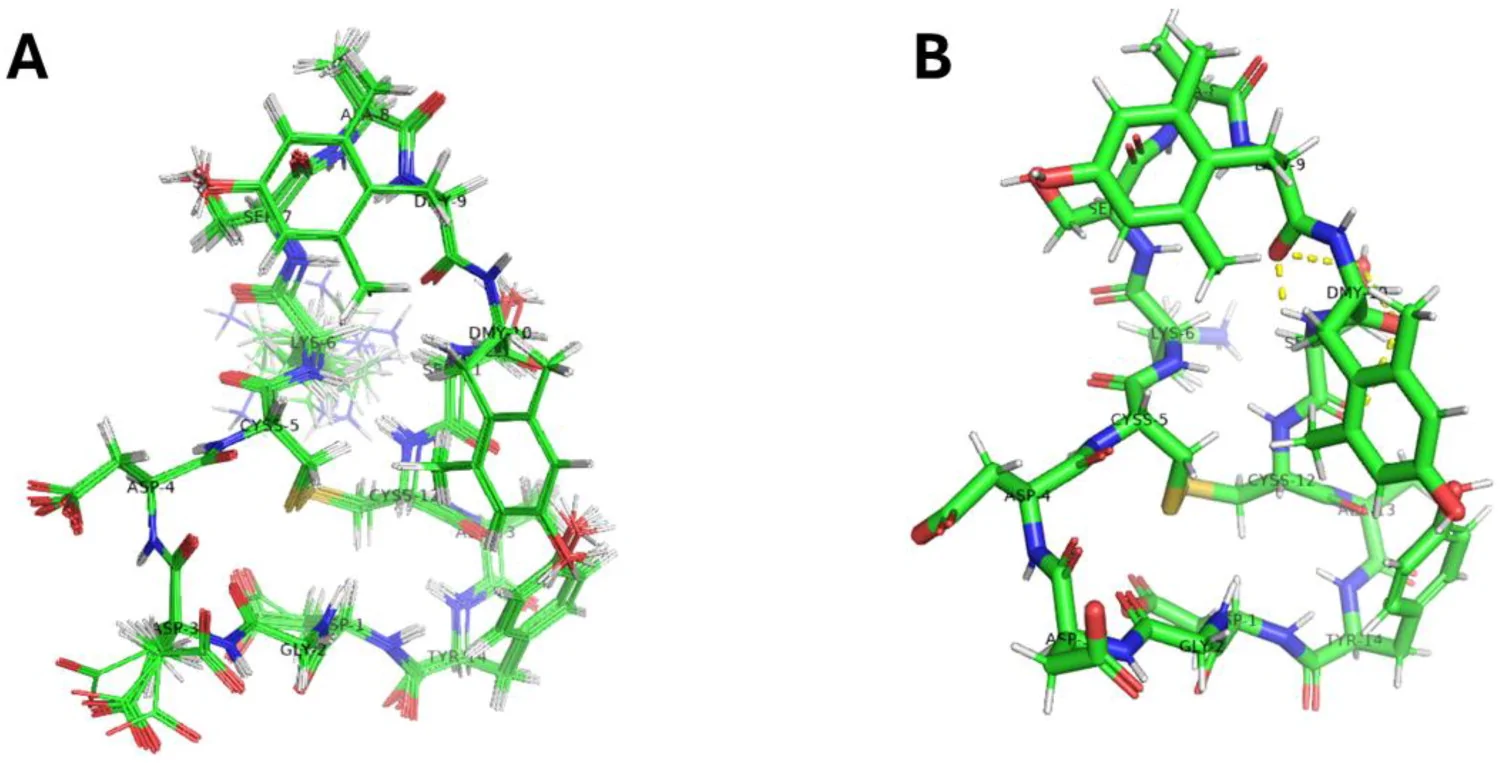
Ensemble of 10 NMR-derived structures using CYANA for (A) SFTI-DMY and (B) one representative structure of SFTI-DMY.

The overall structure of SFT-FGUA was a twisted β-sheet with β-turns at either corner of the β-sheet (Figure 8). The structure of SFTI-DMY was slightly different from that of SFTI-FGUA. SFTI-DMY structure was stabilised by β-turns at G2-D3-D4-C5 and C12, A13, Y14-D1. Thus, one side of the peptide’s disulphide bond formed a β-sheet with turns^31^. The peptide has a twisted backbone structure from K6 to S11 (Figure 9).

**Figure 10.**
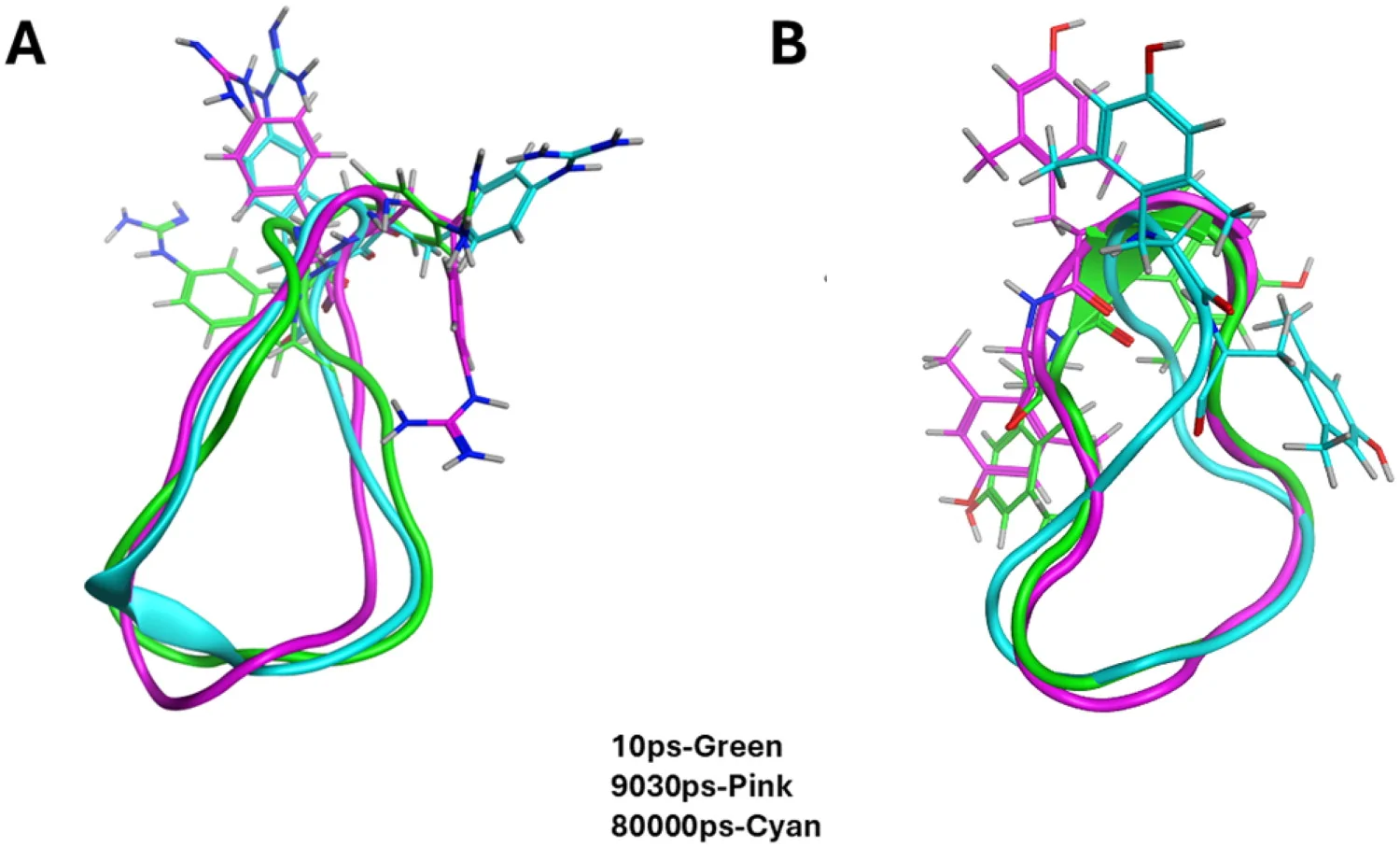
MD simulated structures of (A) SFTI-FGUA and (B) SFTI-DMY at different time lapses during 100 ns dynamics. The time points chosen to represent peptide structures from the backbone RMSD graph where structures exhibited some flexibility during MD simulation.

SFTI-FGUA and SFTI-DMY structures obtained from NMR were subjected to MD simulation without NMR constraints in the presence of water for 100 ns to evaluate the stability of the structure. Overall backbone structure of SFTI-FGUA and disulphide bond seems to be stable during the MD simulations, as indicated by backbone RMSD, dihedral angles of disulphide bond, and Cα-Cα distance of Cys5-Cys12 residues (Figures 10 and S28–S30). However, DMY exhibited change in disulphide bond dihedral angle around 45000 ps with change from +90^º^ to −90° (Figure S28B).

### A molecular model of peptide CD58 interactions

**Figure 11.**
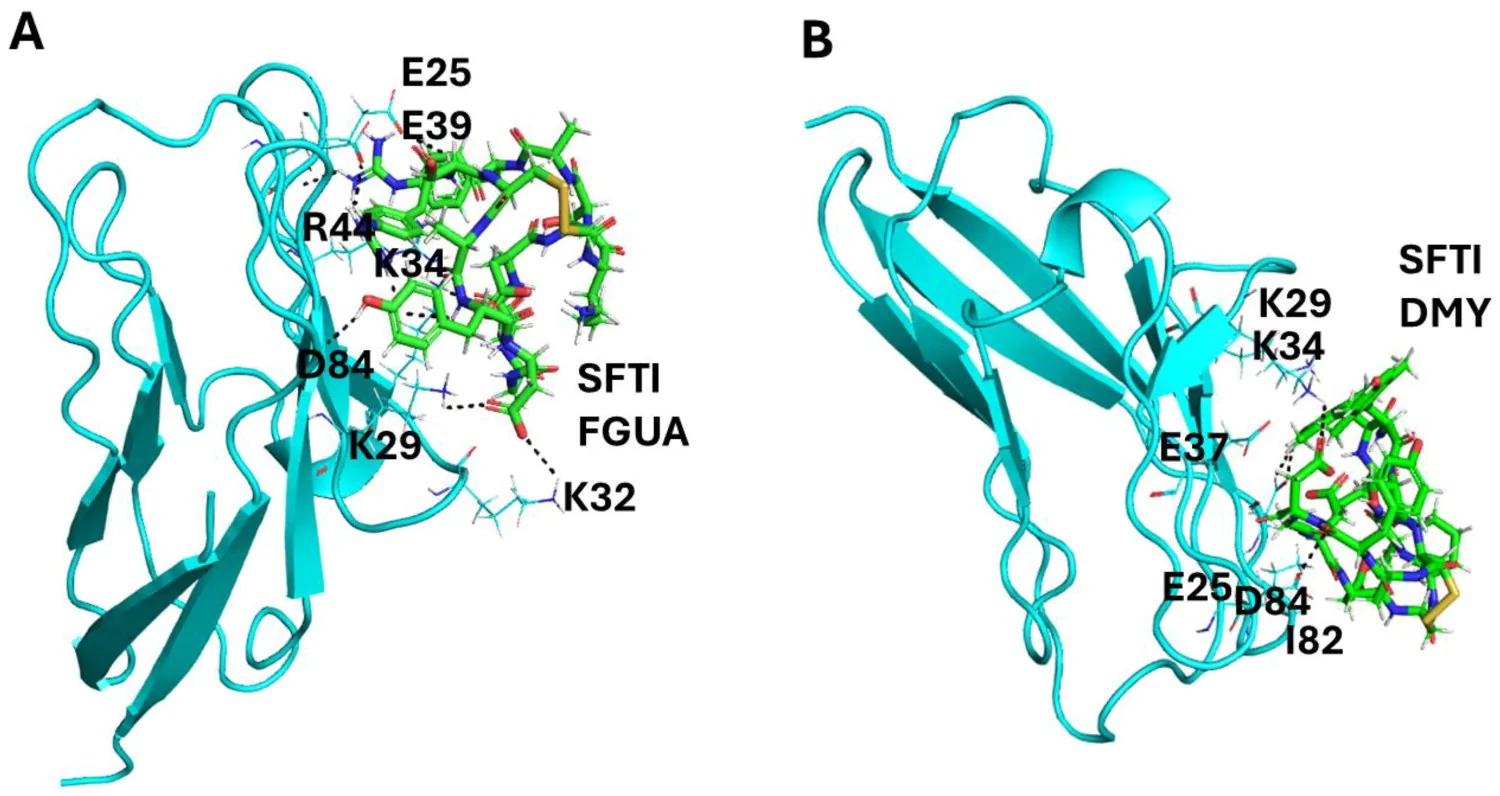
Low-energy docked structures of SFTI-FGUA and SFTI-DMY on CD58: (A) SFTI-FGUA, (B) SFTI-DMY. Molecular Operating Environment (MOE, Chem Computing Group LLC, Canada) software was used for docking. Amino acid residues from the protein are labelled with a single-letter code for amino acids. Ligand atoms are labelled in colours (sticks): carbon-green, nitrogen-blue, oxygen-red, and disulphide bond-yellow.

To model how aptamer peptides designed interact with CD58 and inhibit the CD2-CD58 interaction, we performed docking studies using MOE. The lowest energy docked structures of SFTI-FGUA and SFTI-DMY are shown in Figure 11. The SFTI-FGUA structure was docked on the adhesion domain of CD58. Details of the interaction of the lowest-energy docked structure (docking score of −8.5, Table S4) with CD58 indicated that SFTI-FGUA interacts with CD58 via hydrogen bonding and hydrophobic interactions. Asp1 of SFTI-FGUA formed an electrostatic interaction with the K32 and K29 side chains of CD58; Asp3 of SFTI-FGUA formed an electrostatic interaction with the K34 side chain of CD58. Hydrogen bonding was observed between Tyr14 of the SFTI-FGUA side chain and E78 of CD58, Fgua10 of SFTI-FGUA and E78 of CD58 and the Fgua10 backbone NH of SFTI-FGUA and E25 side chain of CD58. The Fgua9 side chain formed three hydrogen bonds with the E43 and E37 side chains of CD58. The Fgua10 phenyl group formed a hydrophobic interaction with L27 of CD58. In the crystal structure of the CD2-CD58 complex and in mutation studies, it was reported that E25, K29, K32, D33, K34, and E27 are important for binding to CD2^7^. Our docking studies indicated that K29, K32, K34, and E37 of CD58 are involved in binding to SFTI-FGUA.

In the SFTI-DMY docked structure (Table S5), hydrogen bonding was observed between the side chains of Dmy9 and D84 of CD58, and between the Tyr14 backbone of SFTI-DMY and E15 of CD58. The Asp1 side chain of the DMY peptide formed an electrostatic interaction with K34. Dmy9 aromatic group formed a cation-π interaction with K87 of CD58, Dmy10 aromatic group formed a hydrophobic interaction with L27. Compared to SFTI-FGUA, SFTI-DMY formed fewer interactions with the CD58 adhesion domain. These residues are essential for CD2 binding, as highlighted in the CD2-CD58 crystal structure^7^. The possible interaction of SFTI-FGUA and SFTI-DMY for inhibiting CD2-CD58 interaction is shown in Figure S31A,B in the supporting information.

### Binding of peptide to CD58 protein by SPR

We hypothesised that SFTI-DMY and SFTI-FGUA inhibit the CD2-CD58 interaction by binding to the CD58 adhesion domain. To verify the binding, we immobilised the CD58 protein on a CM5 chip, and the peptides SFTI-FGUA and SFTI-DMY were used as analytes. The binding kinetics of CD58-SFTI-FGUA suggested a concentration-dependent binding of SFTI-FGUA to CD58 protein, as seen by the SPR sensorgram (Figure S32A). Based on global binding analysis SFTI-FGUA binds with a K_d_ of 25 µM. When SFTI-DMY was used as an analyte, there was a concentration-dependent binding to CD58 protein, and the K_d_ value obtained was 22 µM (Figure S32B).

### Thermal stability

SFTIs and other grafted peptides are recognised for their enhanced thermal stability compared to nongrafted variants^47^^,^^48^. Thermal stability of SFTI-FGUA and SFTI-DMY was monitored using changes in CD spectra at varying temperatures. CD spectra of these peptides were recorded in water at varying temperature from 25 to 65 °C. The spectra showed minimal alteration with increasing temperature, suggesting a stable peptide conformation in solution (Figure S33). Though CD spectra are adept at detecting conformational alterations, they may not reflect peptide degradation due to temperature or changes in disulphide bonds. To further verify peptide stability, high-performance liquid chromatography (HPLC) analysis was performed after incubation at 55 °C and 85 °C. The results showed no significant variation in HPLC peak area, indicating peptide stability at these elevated temperatures. Moreover, mass spectrometric analysis of these peptides incubated at various temperatures in solution confirmed that the peptide remained intact, without changes in molecular mass (Figures S34 and S35). This evidence suggests that SFTI-FGUA and SFTI-DMY possess resistance to thermal degradation.

Disulphide bonds are well documented for imparting structural rigidity and enhancing the stability of grafted peptides, a strategy that is beneficial for the rational design of stable, effective peptides for drug development^49^. Peptides stability was also assessed by incubating peptides with 0.1, 1 and 5 mM of dithiothreitol. The CD and mass spectrometry data indicated that the peptides maintained stability at both 0.1 and 1 mM concentrations of dithiothreitol (Figures S36–S38). Notably, dithiothreitol at 5 mM could potentially disrupt disulphide bonds, as evidenced by changes in the CD spectra and the emergence of an additional peak with an increase in mass corresponding to two hydrogens.

### Simulated gastric/intestinal fluid stability study

**Figure 12.**
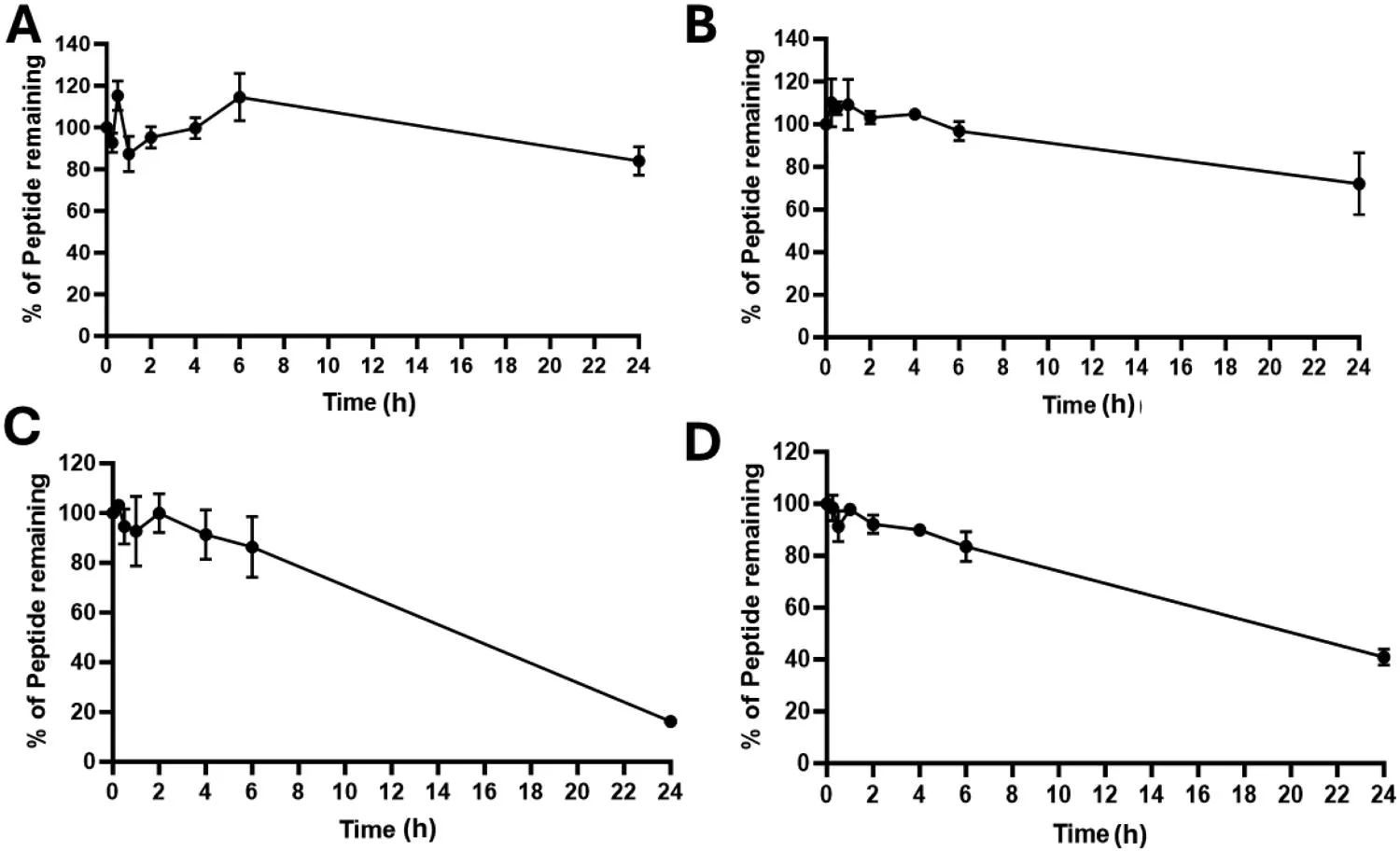
Stability studies in SGF and in human liver microsomes (s9 fraction). (A & B) in simulated gastric fluid for SFTI-FGUA and SFTI-DMY, respectively, (C & D) in human liver microsomes for SFTI-FGUA and SFTI-DMY, respectively. Data are represented as mean ± SD

One significant limitation of using peptides for oral administration is their susceptibility to degradation within the gastrointestinal tract. The acidic nature of gastric juices is a primary factor contributing to the instability of peptide drugs. Furthermore, enzymes such as pepsin, trypsin, chymotrypsin, elastase, and various brush-border membrane-bound enzymes are responsible for the hydrolysis of specific peptide bonds^50^. Therefore, it is essential to evaluate the stability of peptides in both gastric and intestinal fluids in the presence of these enzymes. In this study, we investigated the stability of grafted peptides in simulated gastric fluid (SGF) and simulated intestinal fluid (SIF), taking enzymatic activity into account. SFTI-FGUA and DMY were incubated in SGF for 24 h at 37 °C to determine their enzymatic stability in gastrointestinal fluids. In the case of SFTI-FGUA overall, the peptide exhibited stability for more than 6 h, and there was a slight decrease in the amount of intact peptide. Similarly, the SFTI-DMY peptide remained stable for more than 6 h and, even at 24 h, nearly 80% of the peptide remained intact. The results indicate that both SFTI-FGUA and DMY remained stable over 6 h in simulated gastric fluid (Figures 12A,B, S39, and S40).

When SFTI-FGUA was incubated in simulated intestinal fluid and HPLC analysis was done, the peptide peak was observed at 0 h incubation. The peptide seems to degrade within 15 min, and after 60 min, no peptide peak was observed. Mass spectrometry analysis of the peptide in SIF confirmed the presence of intact peptide up to 24 h. However, the peak intensity diminished drastically after 15 min compared to 0 h. In the case of SFTI-DMY, the HPLC peak intensity diminished within 15 min, and thereafter (at 30 and 60 min) no HPLC peak for the peptide was observed. Mass spectrometry analysis of the sample indicated the presence of an intact peptide up to 24 h for SFTI-FGUA and less than 15 min for SFTI-DMY (Figures S41–S45).

### Liver microsome (HLM-S9)

**Figure 13.**
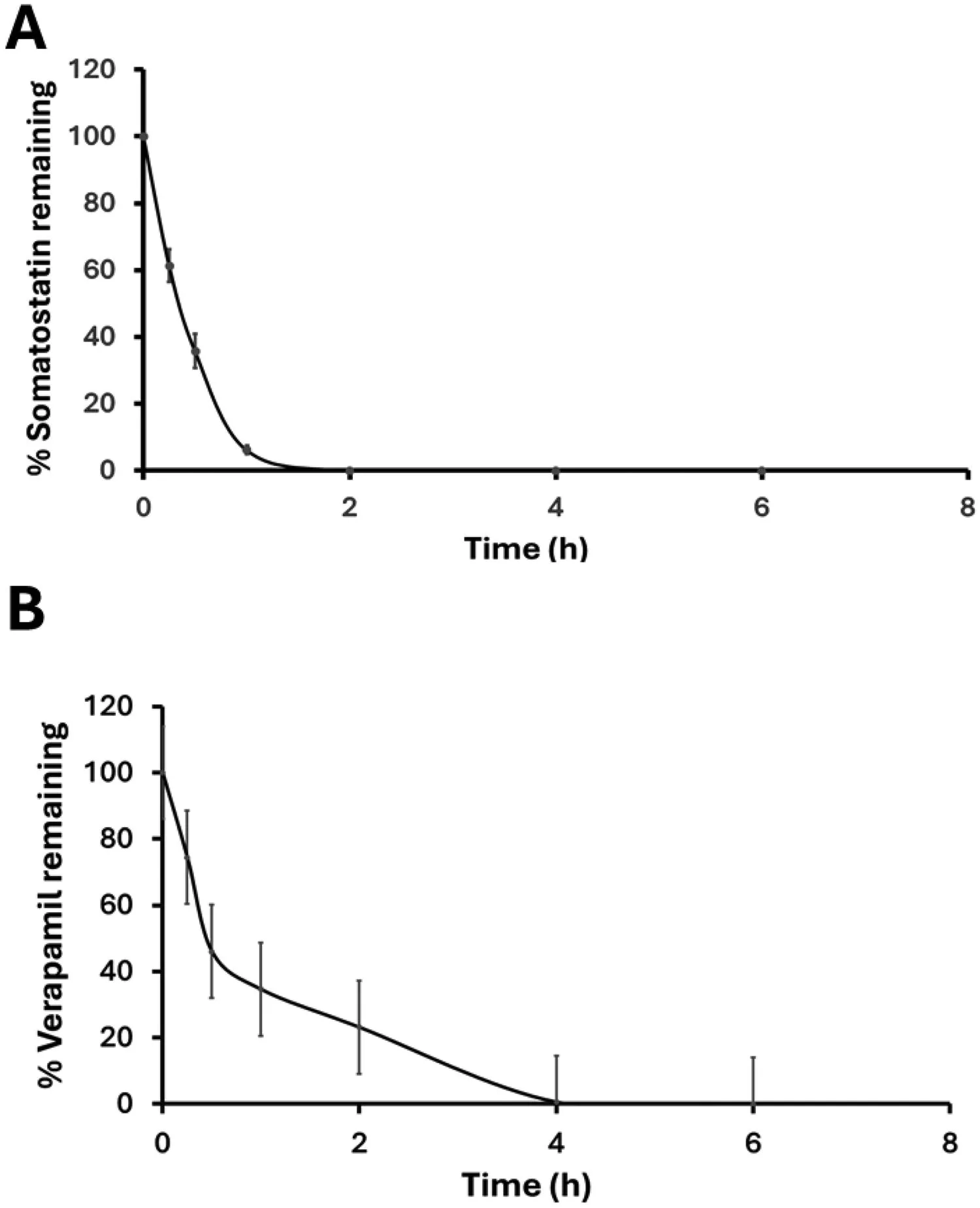
(A) Stability of control peptide in simulated gastric fluid (SGF). At different time points (0, 15 min, 30 min, 1, 2, 4, 6 & 24 h), the extracted peptide was analysed by HPLC. (B) Stability of verapamil (control) in human liver microsomes. Verapamil (Concentration 200 µM) was incubated in human liver microsomes. At different time points (0,15, 30 min, 1, 2, 4, 6, and 24 h), the sample was analysed by HPLC. Data are represented as mean ± SD

The liver serves as the primary site for drug metabolism, with nearly 60% of marketed drugs undergoing cytochrome P450 (CYP)-mediated biotransformation^51^. To assess hepatic metabolism and the potential for first-pass effect during oral administration, the stability of SFTI-a1 was evaluated in liver microsomes^52^ (Corning). Peptide degradation was observed in the presence of liver microsomal enzymes (Figures 12C,D, S46, and S47). After a 6-h incubation, both SFTI-FGUA and DMY retained 80% of their original amounts. The presence of the peptide was further confirmed by mass spectrometry. The precise mechanism of degradation of these peptides were not elucidated by mass spectrometry, as most liver enzymes are cytochromes that primarily facilitate oxidation–reduction and hydroxylation processes. Notably, deamidating enzymes have been identified in liver S9 fractions^53^. The retention of a significant portion of the peptide after 6 h suggests that it is not subject to rapid phase I hepatic metabolism, indicating a potential for limited first-pass metabolism. While this study sheds light on the peptide’s metabolic profile in the liver, it does not provide conclusive data on the oral bioavailability of SFTI-FGUA and DMY. The systemic circulation of orally administered drugs depends on intestinal absorption, which is influenced by the compound’s lipophilicity and overall molecular structure^53^. For stability studies, peptide^39^^,^^54^, somatostatin, and the small-molecule verapamil were used as controls. Verapamil has a calculated half-life of approximately 30 min, as reported in the literature^55^. Controls used for stability in gastric fluid (somatostatin) and liver microsomes (verapamil) are shown in Figure 13A,B.

## Discussion

Aptamer peptides are constrained peptide sequences derived from proteins designed to target protein surfaces for modulation of protein-protein interactions and as possible therapeutic agents. In our study, we used peptide sequences from the CD2 protein to inhibit CD2-CD58 interactions. Peptides derived from CD2 have been shown to bind to CD58 and inhibit the CD2-CD58 interaction, thereby modulating the immune response^8^^,^^11^. However, some of the designed peptides exhibited multiple conformations or were insoluble in water. In this work, functional groups were introduced into the aptamer peptide to restrict the conformational isomerism induced by the proline-proline sequence, using aromatic groups with ring substitutions. Two aptamer peptides were designed, SFTI-FGUA and SFTI-DMY, where FGUA peptide has a guanidine group on the side chain for solubility and bulky groups on the phenyl ring restrict the conformation around Fgua-Fgua sequence. In the SFTI-DMY peptide, the tyrosine hydroxyl group aids in solubility, and the dimethyl groups restrict the conformation of the ring rotation, and the Dmy-Dmy sequence in the peptide restricts the conformation in the backbone (Figures 1 and 2). Conformationally constrained SFTI-1 sequences and peptide with disulphide bond replaced by pyrazole moiety have been reported by other researchers^56^^,^^57^. Both SFTI-FGUA and SFTI-DMY exhibited solubility in water compared to SFTI-DBF (Figures 1F and S1), suggesting that our design concept, with soluble functional groups on the aromatic ring, enhances solubility (Figure 1).

Our intention was not only to enhance the aptamer’s aqueous solubility but also to reduce the multiple conformations of the SFTI framework. SFTI-1 is a stable structure known to tolerate temperature variation and be stable against enzymatic degradation. However, SFTI-1 exhibits multiple conformations in solution due to three prolines in its structure, as X-Pro and Pro-Pro sequences are known to undergo cis-trans isomerisation. We replaced the Pro-Pro sequence in the SFTI framework with the CD2 protein sequence (adhesion strands F and C) with Fgua and Dmy. Detailed NMR studies indicated that both peptides exhibit a single major conformation in solution, with one set of peaks for each amino acid in the sequence. A minor conformation was detected using NMR; however, it was less than 7% of the major conformation as quantified by 1D NMR spectra. Furthermore, the NH-CαH coupling constants and well-resolved amide resonances indicated that both peptides exhibit a single major conformation in solution. The peptide structures derived from NMR distance constraints and CYANA both indicated beta-turns. 3D structures of the two peptides were different, with SFTI-FGUA exhibiting two β-turns around D1-G2-D3-D4 and S7-A8-FGua9-Fgua10 (Figure 8). The γ-turns were around A13-Y14-D1 and C5-K6-S7, whereas for SFTI-DMY, β-turns were observed at G2-D3-D4-C5 and C12, A13, Y14-D1 (Figure 9). Structural flexibility of the NMR structures for both peptides was further evaluated by MD simulations without NMR constraints. The overall backbone structure of SFTI-FGUA remained the same during 100 ns of dynamics. The backbone RMSD, disulphide bond dihedral angles, and Cα-Cα distances around Cys residues remained stable during the dynamics (Figure S28). For SFTI-DMY, the RMSD of the backbone atoms was flexible and changed up to 6 Å compared to SFTI-FGUA from the starting structure. The dihedral angle of the disulphide bond for SFTI-FGUA was around −70°, whereas for SFTI-DMY it was +90°. Most disulphides have either a left-handed spiral conformation (χ1=-60^º^, χ^2^= −90^º^, χ3= −90^º^, χˈ_2_= −90^º^, χˈ_1_= −60^º^) or a right-handed hook conformation (χ_1_=-60^º^, χ_2_= +120^º^, χ_3_= +90^º^, χˈ_2_=-50^º^, χˈ_1_= −60^º^). Due to rigid torsion and favoured disulphide bond geometries, the Cα distances between the linked cysteine residues are typically restrained under 6.5 Å^58^. However, after 45000 ps dynamics the dihedral angle of the disulphide bond for SFTI-DMY was changed to −90°. Even though the NMR constrained structure of SFTI-DMY exhibited different conformation of disulphide bond, MD simulations suggested that the peptide can acquire left-handed conformation. SFTI-1 peptide with three prolines and a disulphide bond has shown multiple conformations in NMR^59^. In the present case SFTI-1 was grafted and modified to remove proline amino acid in the sequence and hence major conformation was observed in NMR. We cannot rule out the possibility of fast conformational changes around Cys residues.

The designed aptamer peptides inhibited adhesion between Jurkat T cells and sRBC, suggesting that the observed adhesion inhibition was due to CD2-CD58 inhibition. SPR studies suggested that SFTI-FGUA and DMY bind to CD58 protein immobilised on the sensor chip (Figure S32). Both peptides inhibit cell adhesion at nanomolar concentrations. However, SPR studies indicate that SFTI-FGUA and SFTI-DMY bind to CD58 with K_d_ value in the micromolar range. SPR studies are conducted with pure CD58 protein that has only D1 domain immobilised using free amine groups. When protein is immobilised on the CM5 chip surface, any free amine can be coupled to the dextran layer of the SPR chip, and hence they have a different orientation. In cells, the full functional protein CD58 has two domains (D1-D2), and proteins are anchored to the membrane via the transmembrane domain. Hence, we may observe different bindings in SPR. Furthermore, what we measure from the E-rosetting assay is the inhibition of adhesion between CD2 and CD58, which is weak and around 1–10 µM^60^^,^^61^. As an orthogonal assay, we studied peptide binding to CD58 on the cell surface using OVCAR-3 cells as a model. Competitive binding assay indicated that both peptide SFTI-FGUA and DMY competitively bind to CD58 on the cell surface at a concentration of less than 1 µM. The peptide’s stability was studied under various conditions, including thermal stability, disulphide-bond stability, and stability in simulated gastric and intestinal fluids, as well as in liver microsomes. Both peptides remained stable in gastric fluid for up to 24 h and in human liver microsomes for up to 6 h, although peptides do not undergo major biotransformation in human liver microsomes, except for side chains. Since the peptide was modified with guanidine and dimethyl groups, stability was assessed in liver microsomes (Figure 12). At 6 h, 80% of the peptides remained intact; by 24 h, most had degraded, with less than 20% intact. Stability data from SGF and human liver microsomes were analysed by fitting the curve to different models using DDSolver^42^. The percentage of peptide degradation was fitted to zero-, first-, and second-order kinetic models. For SFTI-FGUA in SGF, none of the models provided a good fit, whereas for SFTI-DMY in SGF, second-order kinetics provided the best fit. For liver microsome studies, SFTI-FGUA stability data fit zero-order kinetics, whereas for SFTI-DMY, the best fit was first-order kinetics (Figures S48–S51). In intestinal fluid, degradation of both peptides was rapid, and HPLC and mass spectrometry data showed the presence of intact peptide up to 2 h for SFTI-FGUA and at least 15 min for SFTI-DMY (Figures S41–S45). Most peptides degrade rapidly in intestinal fluids, with some degrading within 5 min, whereas cyclic and constrained peptides exhibit stability for 6 to 10 h. Published results on the SFTI peptide with one disulphide bond and backbone cyclisation in SIF indicated a t_1/2_ of around 16 h, whereas the RTD-1 peptide with three disulphide bonds and backbone cyclisation exhibits a t_1/2_ of 3 min. A well-known peptide, somatostatin, exhibits a t_1/2_ of less than 3 min^40^. Pancreatin in the SIF contains the proteases trypsin, chymotrypsin, and elastase, which are largely responsible for the intestinal breakdown of peptide molecules^39^. Thus, we expect peptides to be readily degraded in the intestine. However, the SFTI framework is supposed to stabilise the peptide and make it stable against proteases.

Both SFTI-FGUA and SFTI-DMY peptides inhibited cell adhesion between sheep blood cells and Jurkat T cells at nanomolar concentrations (Figure 3), possibly by blocking the CD2-CD58 interaction. Furthermore, neither peptide exhibited any toxicity in Jurkat T cells, CD58-expressing OVCAR-3 cells, or HFLS-RA cells, suggesting that the peptides exert their effect only on surface proteins involved in cell adhesion inhibition and do not cause antiproliferative effects. To confirm the oral bioavailability of the peptides, their systemic effects in circulation, and their immunomodulatory effects, *in vivo* studies are needed. We are currently working on a collagen-induced arthritis model of arthritis, an autoimmune disease model, to evaluate the oral bioavailability of the peptide and its effect in reducing the immune response.

## Conclusion

Aptamer peptides targeting the CD2 protein epitope were designed by grafting the CD58-binding domain of CD2 onto the SFTI-1 framework. The Pro-Pro sequence in the peptide was replaced with a phenylalanine residue modified with a guanidino group on the aromatic phenyl ring. Another peptide with a similar sequence was grafted onto the SFTI-1 framework by replacing Pro-Pro with dimethyl tyrosine. These peptides exhibited aqueous solubility and a major conformer in solution, as shown by NMR. Detailed NMR structural analysis indicated that the peptides adopt a stable structure in solution. These peptides inhibited cell-cell adhesion between Jurkat cells and sheep red blood cells, indicating inhibition of the CD2-CD58 interaction. Peptides also exhibited stability against changes in temperature, gastric fluid, and human liver microsomes. Taken together, these studies suggest that stable peptides can be designed to target autoimmune diseases.

## Supplementary Material

SupportingInformation (1).docx

## Data Availability

Most of the data is available as supplementary material. Additional data is available from the authors in accordance with the University Guidelines.
