# Supplementary material for: Design of aptamer peptides for immunomodulation
Source: J Enzyme Inhib Med Chem. 2026 Sep 2;41(1):2717480. doi: 10.1080/14756366.2026.2717480 (PMC13539786; doi:10.1080/14756366.2026.2717480)
Supplement: Supplementary file 1 [file IENZ_A_2717480_SM9828.docx]

**Supporting Information**

**Table S1.** The sequence of aptamer peptides

| Peptide name | Sequence |
| --- | --- |
| SFTI-1 | Cyclo(G^1^R^2^C^3^T^4^K^5^S^6^I^7^P^8^P^9^I^10^C^11^F^12^P^13^D^14^) |
| SFTI-FGUA | Cyclo (D^1^G^2^D^3^D^4^ C^5^K^6^S^7^A^8^-Fgua^9^-Fgua^10^-S^11^C^12^ A^13^Y^14^) |
| SFTI-DMY | Cyclo (D^1^G^2^D^3^D^4^ C^5^K^6^S^7^A^8^-Dmy^9^-Dmy^10^-S^11^C^12^ A^13^Y^14^) |
| SFTI-FGUA-6FAM | Cyclo (D^1^G^2^D^3^D^4^ C^5^K^6^S^7^A^8^-Fgua^9^-Fgua^10^-S^11^C^12^ A^13^Y^14^)  K6 side chain was attached to 6-FAM |

**Table S2.** Chemical shift and coupling constant of amide resonance of ^1^H NMR resonances for peptide SFTI-FGUA in H_2_O (90%) / D_2_O (10%). NMR data were collected on a Bruker 600 MHz spectrometer. Chemical shift and coupling constants are reported for spectra recorded at 298K

.

| AA | ^H^ | HA | HB2/QB | HB3 | QD | HH | QE | QG | QZ | CA | CB | CD | CE | CG | N | ^3^J_HH_ |
| --- | --- | --- | --- | --- | --- | --- | --- | --- | --- | --- | --- | --- | --- | --- | --- | --- |
| D1 | 8.181 | 4.528 | 2.719 | 2.785 |  |  |  |  |  | 50.44 | 35.77 |  |  |  | 119.2 | 7.46 |
| G2 | 8.012 | 3.902  3.828 |  |  |  |  |  |  |  | 43.04 |  |  |  |  | 108.4 | 6.21 |
| D3 | 8.199 | 4.564 | 2.777 |  |  |  |  |  |  | 50.61 | 35.75 |  |  |  | 121.3 | 4.72 |
| D4 | 8.417 | 4.588 | 2.711 | 2.853 |  |  |  |  |  | 50.74 | 35.58 |  |  |  | 118.2 | 7.46 |
| C5 | 8.093 | 4.534 | 3.007 | 3.117 |  |  |  |  |  | 53.66 | 38.61 |  |  |  | 118.9 | 6.98 |
| K6 | 8.370 | 4.239 | 1.701 | 1.822 | 1.586 |  | 3.122  3.011 | 1.383  1.317 | 7.427 | 54.32 | 29.87 | 26.28 | 39.38 | 22.15 | 123.3 | 7.04 |
| S7 | 7.828 | 4.335 | 3.692 | 3.778 | 3.784  3.687 |  |  |  |  | 55.35 | 61.16 |  |  |  | 114.4 | 7.41 |
| A8 | 8.146 | 4.097 | 1.112 |  |  |  |  |  |  | 50.38 | 16.02 |  |  |  | 124.9 | 5.95 |
| Fgua9 | 8.037  7.161 | 4.385 | 2.881  3.079 | 3.078 |  |  |  |  |  | 55.57 | 35.72 |  |  |  | 115.0 | 3.38 |
| Fgua10 | 7.958  7.276 | 4.500 | 3.039  3.042 | 3.137 |  |  |  |  |  | 55.27 | 36.54 |  |  |  | 118.1 | 3.72 |
| S11 | 8.044 | 4.305 | 3.753 |  |  |  |  |  |  | 55.58 | 60.69 |  |  |  | 117.1 | 3.58 |
| C12 | 8.021 | 4.571 | 3.029 | 3.036 |  |  |  |  |  | 52.81 | 39.09 |  |  |  | 118.9 | 4.88 |
| A13 | 8.323 | 4.100 | 1.199 |  |  |  |  |  |  | 50.20 | 16.23 |  |  |  | 125.4 | 5.36 |
| Y14 | 7.815 | 4.429 | 2.906 | 2.998 | 7.011 |  |  |  |  | 55.17 | 35.78 |  |  |  | 117.2 | 7.91 |

**Table S3.** Chemical shift and coupling constant of amide resonance of ^1^H NMR resonances for peptide SFTI-DMY in H_2_O (90%) / D_2_O (10%). NMR data were collected on a Bruker 600 MHz spectrometer. Chemical shift and coupling constants are reported for spectra recorded at 298K.

| AA | ^H^ | HA | HB2/QB | HB3 | QD | HH | QE | QG | QZ | CA | CB | CD | CE | CG | N | ^3^J_HH_ |
| --- | --- | --- | --- | --- | --- | --- | --- | --- | --- | --- | --- | --- | --- | --- | --- | --- |
| D1 | 8.105 | 4.556 | 2.705 | 2.797 |  |  |  |  |  | 50.41 | 35.32 |  |  |  | -- | -- |
| G2 | 8.040 | 3.829  3.891 |  |  |  |  |  |  |  | 43.04 |  |  |  |  | 108.6 | 5.87 |
| D3 | 8.162 | 4.596 | 2.734 | 2.803 |  |  |  |  |  | 50.64 | 35.84 |  |  |  | 118.7 | 6.84 |
| D4 | 8.387 | 4.605 | 2.713 | 2.893 |  |  |  |  |  | 50.59 | 35.40 |  |  |  | 118.0 | 6.61 |
| C5 | 8.105 | 4.537 | 3.016 | 3.086 |  |  |  |  |  | 52.72 | 38.76 |  |  |  | 118.9 | 6.76 |
| K6 | 8.430 | 4.274 | 1.695 | 1.829 | 1.584 |  | 2.901 | 1.339 | 7.418 | 55.95 | 29.96 | 26.25 | 39.44 | 22.11 | 123.9 | 7.15 |
| S7 | 7.792 | 4.227 | 3.557 | 3.760 | 3.838 |  |  |  |  | 55.20 | 61.15 |  |  |  | 114.1 | 7.40 |
| A8 | 8.219 | 4.172 | 1.161 |  |  |  |  |  |  | 50.14 | 15.92 |  |  |  | 125.1 | 5.54 |
| Dmy9 | 8.099  6.487 | 4.316 | 2.880  2.173 | 2.118 |  |  |  |  |  | 54.35 | 19.55 |  |  |  | 118.8 | 6.76 |
| Dmy10 | 7.762  6.522 | 4.256 | 3.099  2.155 | 2.984  2.182 |  |  |  |  |  | 54.26 | 29.96 |  |  |  | 118.1 | 6.05 |
| S11 | 7.858 | 4.386 | 3.807 | 3.886 |  |  |  |  |  | 55.48 | 61.33 |  |  |  | 114.7 | 7.41 |
| C12 | 7.945 | 4.535 | 2.959 | 3.006 |  |  |  |  |  | 53.61 | 38.83 |  |  |  | 120.5 | 6.93 |
| A13 | 8.143 | 3.988 | 1.117 |  |  |  |  |  |  | 50.92 | 16.03 |  |  |  | 124.9 | 4.74 |
| Y14 | 7.746 | 4.418 | 2.875 | 2.979 | 6.982 |  |  |  |  | 55.23 | 35.55 |  |  |  | 116.6 | 6.78 |

**Table S4**. Docking energy of low-energy structures of SFTI-FGUA to the CD58 protein evaluated by MOE

| **Rank** | **Binding free energy (London dG Score)** |
| --- | --- |
| 1 | -8.5818 |
| 2 | -8.0457 |
| 3 | -7.7465 |
| 4 | -7.4312 |
| 5 | -7.4290 |

**Table S5**. Docking energy of low-energy structures of SFTI-DMY to the CD58 protein evaluated by MOE.

| **Rank** | **Binding free energy (London dG Score)** |
| --- | --- |
| 1 | -7.8102 |
| 2 | -6.7342 |
| 3 | -6.0689 |
| 4 | -5.7749 |
| 5 | -5.7680 |

**Figure S1**. Solubility of aptamer peptides at different peptide concentrations (solubility at higher concentrations is provided in the main document). A) water, and SFTI-DBF, DMY, and FGUA at 3mg/mL. B) SFTI-FGUA and C) SFTI-DMY in water at different concentrations.

**Figure S2**. HPLC chromatogram for peptide SFTI-FGUA.

**Figure S3**. MALTI-TOF mass spectrum for SFTI-FGUA

**Figure S4**. HPLC chromatogram for peptide SFTI-DMY

**Figure S5**. MALDI-TOF mass spectrum for SFTI-DMY

**Figure S6.** Structure of SFTI-FGUA-6FAM

**Figure S7.** HPLC Chromatogram of SFTI-FGUA-6FAM

**Figure S8.** MALDI-TOF mass spectrum of SFTI-FGUA-6FAM

**Figure S9**. Representative pictures of the E-rosetting assay for peptide SFTI-FGUA. Image was captured using Nikon Eclipse T2S, Objective magnification: 20X/ N.A. 0.4, Scale bar: 50μm, Expanded regions of images are also shown for E-rosetting.

**Figure S10A**. Representative pictures of the e-rosetting assay for peptide SFTI-DMY. E-rosetting inhibition at 25 and 12.5 nM.. Image was captured using Nikon Eclipse T2S, Objective magnification: 20X/ N.A. 0.4, Scale bar: 50 μm, Expanded regions of images are also shown for E-rosetting

**Figure S10B**. Representative pictures of the e-rosetting assay for peptide SFTI-DMY. E-rosetting inhibition at 6.25 and 1.56 nM. Image was captured using Nikon Eclipse T2S, Objective magnification: 20X/ N.A. 0.4, Scale bar: 50 μm, Expanded regions of images are also shown for E-rosetting

**Figure S11**. Representative pictures of the e-rosetting assay without peptide. Image was captured using Nikon Eclipse T2S, Objective magnification: 20X/ N.A. 0.4, Scale bar: 50μm.

**Figure S12**. Expression of CD58 on OVCAR-3 cells verified by flow-cytometry using phycoerythrin (PE)-labeled antibody to CD58 Em λ 576 nm. Data are represented as mean ±S.D.

**Figure S13**. Cell viability assay for OVCAR-3 cells in the presence of SFTI-FGUA and DMY. The viability percentage is shown across different peptide concentrations. Sodium Dodecyl Sulfate (SDS) and media are used as controls. Data are represented as mean ±S.D.

**Figure S14**. Cell viability assay for HFLS-RA cells in the presence of aptamer peptides SFTI-FGUA and SFTI-DMY. Viability is shown at different peptide concentrations. Sodium Dodecyl Sulfate (SDS) and media are used as controls. Data are represented as mean ±S.D.

**Figure S15**. Cell viability assay for Jurkat T cells in the presence of aptamer peptides SFTI-FGUA and SFTI-DMY. Data are represented as mean ±S.D.

**Figure S16**. Analysis of CD spectra of FGUA peptide to obtain components of secondary structures using BestSel. Experimental and calculated CD spectra are shown. The percentage of secondary structure information was obtained from the best-fit spectra. RMS deviation of experimental and calculated CD spectra was 0.0925. Predicted values of beta sheet and beta turn were 22% and 14% with, respectively, with a right-twisted sheet/turn of 14%.

**Figure S17**. Analysis of CD spectra of DMY peptide to obtain components of secondary structures using BestSel. Experimental and calculated CD spectra are shown. The percentage of secondary structure information was obtained from the best-fit spectra. RMS deviation of experimental and calculated CD spectra was 0.6967. Predicted values for beta sheet and beta turn were 20% and 17%, respectively, with a right-twisted sheet/turn of 19%.

**Figure S18**. 2D ^1^H TOCSY spectra for SFTI-FGUA peptide in water. Amino acids in the peptide are labeled with a single-letter code. Fgua is abbreviated as Fg. 1.5 mg of the peptide in 90% H₂O and 10% D₂O.NMR experiments were recorded at 298 K on a Bruker Avance NEO 600 MHz spectrometer

**Figure S19**. 2D ^1^H NOESY spectra for SFTI-FGUA showing NH-CαH region. Amino acids in the peptide are labeled with a single-letter code. Fgua is abbreviated as Fg. 1.5 mg of the peptide in 90% H₂O and 10% D₂O.NMR experiments were recorded at 298 K on a Bruker Avance NEO 600 MHz spectrometer

**Figure S20**. 2D ^1^H DQF COSY for SFTI-FGUA showing NH-CαH region with assignments. 1.5 mg of the peptide in 90% H₂O and 10% D₂O.NMR experiments were recorded at 298 K on a Bruker Avance NEO 600 MHz spectrometer

**Figure S21**. 2D HSQC (^1^H-^13^C) spectra for the SFTI-FGUA peptide. 1.5 mg of the peptide in 90% H₂O and 10% D₂O.NMR experiments were recorded at 298 K on a Bruker Avance NEO 600 MHz spectrometer

**Figure S22**. 2D ^1^H TOCSY spectra for SFTI-DMY peptide in water showing the fingerprint region. 1.5 mg of the peptide in 90% H₂O and 10% D₂O.NMR experiments were recorded at 298 K on a Bruker Avance NEO 600 MHz spectrometer

**Figure S23**. DQF-COSY for SFTI-DMY in water showing NH-CαH region with assignments. 1.5 mg of the peptide in 90% H₂O and 10% D₂O.NMR experiments were recorded at 298 K on a Bruker Avance NEO 600 MHz spectrometer

**Figure S24**. 2D ^1^H NOESY spectra for SFTI-DMY showing NH-CαH region. 1.5 mg of the peptide in 90% H₂O and 10% D₂O.NMR experiments were recorded at 298 K on a Bruker Avance NEO 600 MHz spectrometer

**Figure S25**. 2D HSQC (^1^H-^13^C) spectra for the SFTI-DMY peptide. 1.5 mg of the peptide in 90% H₂O and 10% D₂O. NMR experiments were recorded at 298 K on a Bruker Avance NEO 600 MHz spectrometer

**Figure S26**. ^1^H NH region of 1D NMR spectra of SFTI-FGUA at different temperatures (90% H₂O and 10% D₂O).

**Figure S27**. ^1^H NH region of 1D NMR spectra of SFTI-DMY at different temperatures (90% H₂O and 10% D₂O)

**Figure S28**. Molecular Dynamics (MD) simulations of SFTI-FGUA and DMY structures without NMR constraints for 100 ns MD. NAMD was used for MD simulations with water as solvent. A) variations in backbone rmsd of SFTI-FGUA and DMY, B) variations in dihedral angle of disulfide bond in the structures of SFTI-FGUA and DMY, C) variations in distance between Cα atoms of Cys residues.

**Figure S29**. Dihedral angle phi (φ ) and psi (ψ )variations around possible beta turns in the peptide SFTI-FGUA during MD simulation.

**Figure S30**. Dihedral angle phi (φ ) and psi (ψ )variations around possible beta turns in the peptide SFTI-DMY during MD simulation

**Figure S31**. Low energy docked structure of A) SFTI-FGUA and B) SFTI-DMY with CD58 and overlay of CD2-CD58 complex crystal structure. Overlay of CD2-CD58 complex structure with SFTI-FGUA and DMY docked reveals that SFTI-FGUA and DMY can interfere with CD2-CD58 interaction (PDB ID:1QA9).

**Figure S32**. Binding of peptides to the CD58 protein D1 domain analyzed by surface plasmon resonance (SPR). A Biacore 1K was used to measure binding kinetics. Protein was immobilized on a CM5 chip, and SFTI-FGUA/SFTI-DMY was used as the analyte. Representative sensorgrams of binding kinetics showing the association and dissociation of A) SFTI-FGUA and B) SFTI-DMY.

**Figure S33**. Thermal stability of peptides monitored by circular dichroism spectra A) SFTI-FGUA and B) SFTI-DMY at 25 and 65 °C. Overall, peptide spectra did not change significantly at 65 °C compared to 25 °C.

**Figure S34.** MALDI-TOF mass spectra of SFTI-FGUA sample subjected to high temperatures (70 °C) for 24 h in water.

**Figure S35.** MALDI-TOF mass spectra of SFTI-DMY sample subjected to high temperatures (70 °C) for 24 h in water.

**Figure S36.** Disulfide bond stability of peptides. CD spectra of peptides SFTI-FGUA and DMY in the absence and presence of a reducing agent, dithiothreitol (DTT), at 0.1, 1, and 5 mM of DTT, incubated for 30 min. Peptide concentration was 200 µM. Up to 1 mM of DTT, both peptides showed CD spectra similar to those without DTT. At 5 mM, the CD spectra changed, suggesting a shift in the peptides' overall secondary structure, possibly due to reduced disulfide bonds.

**Figure S37**. Disulfide bond stability of SFTI-FGUA. Mass spectrometry data for SFTI-FGUA in the absence and presence of reducing agent, dithiothreitol (DTT), at 0.1, 1, and 5 mM of DTT, incubated for 30 min

**Figure S38**. Disulfide bond stability of SFTI-DMY. Mass spectrometry data for SFTI-DMY in the absence and presence of reducing agent, dithiothreitol (DTT), at 0.1, 1, and 5 mM of DTT, incubated for 30 min.

**Figure S39.** Stability of SFTI-FGUA in simulated gastric fluid (SGF) using mass spectrometry data (MALDI-TOF). For details, see the text in the main document.

**Figure S40**. Stability of SFTI-DMY in simulated gastric fluid (SGF) using mass spectrometry data (MALDI-TOF). For details, see the text in the main document.

**Figure S41.** Stability of SFTI-FGUA in simulated intestinal fluid (SIF). The peptide was incubated in SIF with relevant enzymes and at different incubation times, the sample was extracted and analyzed by HPLC.

**Figure S42.** Stability of SFTI-FGUA in simulated intestinal fluid (SIF). The peptide was incubated in SIF with relevant enzymes, and at different incubation times, the sample was extracted and analyzed by MALDI-TOF mass spectrometry data

**Figure S43.** Stability of SFTI-FGUA in simulated intestinal fluid (SIF) at 2 and 24 h. The peptide was incubated in SIF with relevant enzymes and at different incubation times, the sample was extracted and analyzed by MALDI-TOF mass spectrometry data

**Figure S44.** Stability of SFTI-DMY in simulated intestinal fluid (SIF). The peptide was incubated in SIF with relevant enzymes, and at different incubation times, the sample was extracted and analyzed by HPLC.

**Figure S45.** Stability of SFTI-DMY in simulated intestinal fluid (SIF). The peptide was incubated in SIF with relevant enzymes, and at different incubation times, the sample was extracted and analyzed by MALDI-TOF mass spectrometry data

**Figure S46.** Stability of SFTI-FGUA in human liver microsomes shown by MALDI-TOF. Peptide (100 µL of 1 mM SFTI-FGUA) was incubated in human liver microsome (pooled liver microsome from Gibco). At different time points (15 and 30 min, 1, 2, 4, 6, and 24 h), 250 µL of the mixture was pipetted into 750 µL of chilled acetonitrile. After extraction and lyophilization, the sample was analyzed by mass spectrometry.

**Figure 47.** Stability of SFTI-DMY in human liver microsomes shown by MALDI-TOF. Peptide (100 µL of 1 mM SFTI-DMY) was incubated in human liver microsome (pooled liver microsome from Gibco). At different time points (15 and 30 min, 1, 2, 4, 6, and 24 h), 250 µL of the mixture was pipetted into 750 µL of chilled acetonitrile. After extraction and lyophilization, the sample was, analyzed by mass spectrometry.

| **Model** | **Adjusted R^2^** | **Mean Squared error (MSE)** | **Equation** | **Slope** | **Half life (hr)** | **Comment** |
| --- | --- | --- | --- | --- | --- | --- |
| **Zero order** | 0.0249 | 127.899 | C_t_ = 101.303−0.5755t | 0.5755 % h^−1^ | 86.9 | Best fit (but poor R^2^) |
| **First order** | 0.0544 | 0.01243 | ln(C_t_)= 4.6143−0.00619t | 0.00619 h^−1^ | 111.9 |  |
| **Second order** | 0.0866 | 1.23×10^−6^ | 1/ C_t_ = 0.009945+6.70×10^-5^t | 6.70×10−5  (%)^−1^ h^−1^ | 149.3 |  |

**Figure 48**. Curve fitting of data from SGF stability studies for SFTI-FGUA. The percentage of peptide degradation was fitted to zero-, first-, and second-order kinetic models in DDSolver. The goodness of fit was assessed using adjusted R^2^ and mean squared error (MSE). The table represents parameters for different models.

| **Model** | **Adjusted R^2^** | **Mean Squared error (MSE)** | **Equation** | **Slope** | **Half life (hr)** | **Comment** |
| --- | --- | --- | --- | --- | --- | --- |
| **Zero order** | 0.9016 | 14.958 | C_t_ =107.3183−1.4629t | 1.4629 % h^−1^ | 34.18 |  |
| **First order** | 0.9240 | 0.001452 | ln (C_t_)=4.6798−0.01657t | 0.01657 h^−1^ | 41.82 |  |
| **Second order** | 0.9391 | 1.51×10^−7^ | 1/ C_t_ =0.009229+0.0001903t | 0.0001903 (%)^−1^ h^−1^ | 52.55 | Best fit |

**Figure 49**. Curve fitting of data from SGF stability studies for SFTI-DMY. The percentage of peptide degradation was fitted to zero-, first-, and second-order kinetic models in DDSolver. The goodness of fit was assessed using adjusted R^2^ and mean squared error (MSE). The table represents parameters for different models.

| **Model** | **Adjusted R^2^** | **Mean Squared error (MSE)** | **Equation** | **Slope** | **Half life (hr)** | **Comment** |
| --- | --- | --- | --- | --- | --- | --- |
| **Zero order** | 0.9715 | 23.19 | C_t_ =102.042−3.4959t | 3.4959  % h^−1^ | 14.03 | Best fit |
| **First order** | 0.9565 | 0.0172 | ln (C_t_) =4.6966−0.07652t | 0.07652 h^−1^ | 9.06 |  |
| **Second order** | 0.9372 | 2.06 X 10^-5^ | 1/ C_t_ =0.006603+0.002184t | 0.002184 (%)^−1^ h^−1^ | 4.58 |  |

**Figure 50**. Curve fitting of data from human liver microsome stability studies for SFTI-FGUA. The percentage of peptide degradation was fitted to zero-, first-, and second-order kinetic models in DDSolver. The goodness of fit was assessed using adjusted R^2^ and mean squared error (MSE). The table represents parameters for different models.

| **Model** | **Adjusted R^2^** | **Mean Squared error (MSE)** | **Equation** | **Slope** | **Half life (hr)** | **Comment** |
| --- | --- | --- | --- | --- | --- | --- |
| **Zero order** | 0.9809 | 7.095 | C_t_ = 97.9824−2.3725t | 2.3725 % h^−1^ | 21.07 |  |
| **First order** | 0.9831 | 0.00148 | ln (C_t_) = 4.6040−0.03651t | 0.03651 h^−1^ | 18.98 | Best fit |
| **Second order** | 0.9728 | 6.46 X 10^-7^ | 1/ C_t_ = 0.009640 + 0.0005975t | 0.0005975  (%)^−1^ h^−1^ | 16.74 |  |

**Figure 51**. Curve fitting of data from human liver microsome stability studies for SFTI-DMY. The percentage of peptide degradation was fitted to zero-, first-, and second-order kinetic models in DDSolver. The goodness of fit was assessed using adjusted R^2^ and mean squared error (MSE). The table represents parameters for different models.

**CYANA library file for the FGUA and DMY**

Numbering schemes used for FGUA and DMY.

```
RESIDUE   FGUA      8   35    3   34

   1 OMEGA    0    0    0.0000    2    1    3    4    0
   2 PHI      0    0    0.0000    1    3    5   33    0
   3 CHI1     0    0    0.0000    3    5    6    7   31
   4 CHI2     0    0    0.0000    5    6    7    8   26
   5 CHI3     0    0    0.0000    9   14   15   16   23
   6 CHI4     0    0    0.0000   14   15   16   17   22
   7 CHI5     0    0    0.0000   15   16   19   20   22
   8 PSI      0    0    0.0000    3    5   33   35    0
   1 C    C_BYL    0    0.0000   -1.0074    1.0939   -0.1000    2    3    0    0    0
   2 O    O_BYL    0    0.0000   -2.0414    0.5866    0.3317    1    0    0    0    0
   3 N    N_AMI    0    0.0000    0.1000    0.3960   -0.3300    1    4    5    0    0
   4 H    H_AMI    0    0.0000    0.8930    0.8574   -0.6746    3    0    0    0    0
   5 CA   C_ALI    0    0.0000    0.1630   -1.0230   -0.0850    3    6   32   33    0
   6 CB   C_ALI    0    0.0000   -0.8910   -1.8190   -0.8700    5    7   29   30    0
   7 CG   C_ARO    0    0.0000   -2.2760   -1.3070   -0.7160    6    8   12    0    0
   8 CD2  C_ARO    0    0.0000   -2.7920   -0.9370    0.5310    7    9   11    0    0
   9 CE2  C_ARO    0    0.0000   -4.0980   -0.4800    0.6630    8   10   14    0    0
  10 HE2  H_ARO    0    0.0000   -4.4620   -0.1730    1.6570    9    0    0    0   27
  11 HD2  H_ARO    0    0.0000   -2.1540   -0.9980    1.4270    8    0    0    0   26
  12 CD1  C_ARO    0    0.0000   -3.1100   -1.2240   -1.8380    7   13   25    0    0
  13 CE1  C_ARO    0    0.0000   -4.4230   -0.7870   -1.7220   12   14   24    0    0
  14 CZ   C_ARO    0    0.0000   -4.9440   -0.4000   -0.4650    9   13   15    0    0
  15 NH   N_AMO    0    0.0000   -6.2390    0.1420   -0.3780   14   16   23    0    0
  16 CQ   C_BYL    0    0.0000   -7.1930   -0.2700    0.6280   15   17   19    0    0
  17 NI3  N_AMO    0    0.0000   -8.3270    0.3530    0.8330   16   18    0    0    0
  18 HI3  H_AMI    0    0.0000   -8.4870    1.1660    0.2810   17    0    0    0    0
  19 NI1  N_AMO    0    0.0000   -6.8230   -1.3430    1.4840   16   20   21    0    0
  20 HI11 H_AMI    0    0.0000   -6.3080   -2.0710    1.0420   19    0    0    0   22
  21 HI12 H_AMI    0    0.0000   -7.5580   -1.6670    2.0750   19    0    0    0   22
  22 QI1  PSEUD    0    0.0000   -6.9330   -1.8690    1.5585    0    0    0    0    0
  23 HH   H_AMI    0    0.0000   -6.6640    0.3110   -1.2670   15    0    0    0    0
  24 HE1  H_ARO    0    0.0000   -5.0520   -0.7360   -2.6250   13    0    0    0   27
  25 HD1  H_ARO    0    0.0000   -2.7260   -1.5180   -2.8270   12    0    0    0   26
  26 QD2  PSEUD    0    0.0000   -2.4400   -1.2580   -0.7000    0    0    0    0   28
  27 QE2  PSEUD    0    0.0000   -4.7570   -0.4545   -0.4840    0    0    0    0   28
  28 QRA  PSEUD    0    0.0000   -3.5985   -0.8562   -0.5920    0    0    0    0    0
  29 HB3  H_ALI    0    0.0000   -0.8390   -2.8810   -0.5050    6    0    0    0   31
  30 HB2  H_ALI    0    0.0000   -0.6010   -1.8400   -1.9570    6    0    0    0   31
  31 QB   PSEUD    0    0.0000   -0.7200   -2.3605   -1.2310    0    0    0    0    0
  32 HA   H_ALI    0    0.0000    0.0100   -1.1960    1.0260    5    0    0    0    0
  33 C    C_BYL    0    0.0000    1.5560   -1.6250   -0.4260    5   34   35    0    0
  34 O    O_BYL    0    0.0000    2.4623   -0.9123   -0.8545   33    0    0    0    0
  35 N    N_AMI    0    0.0000    1.6952   -2.9316   -0.2269   33    0    0    0    0

RESIDUE   DMY      8   33    3   32

   1 OMEGA    0    0    0.0000    2    1    3    4    0
   2 PHI      0    0    0.0000    1    3    5   31    0
   3 CHI1     0    0    0.0000    3    5    6    7   29
   4 CHI2     0    0    0.0000    5    6    7    8   26
   5 CHI3     0    0    0.0000    7    8   11   12   15
   6 CHI4     0    0    0.0000    9   18   19   20   20
   7 CHI5     0    0    0.0000    7   16   22   23   26
   8 PSI      0    0    0.0000    3    5   31   33    0
   1 C    C_BYL    0    0.0000   -1.4818    0.9070    0.6353    2    3    0    0    0
   2 O    O_BYL    0    0.0000   -2.5015    0.3278    1.0065    1    0    0    0    0
   3 N    N_AMI    0    0.0000   -0.3770    0.2650    0.2700    1    4    5    0    0
   4 H    H_AMI    0    0.0000    0.4042    0.7827   -0.0165    3    0    0    0    0
   5 CA   C_ALI    0    0.0000   -0.3000   -1.1710    0.2850    3    6   30   31    0
   6 CB   C_ALI    0    0.0000   -1.3810   -1.8550   -0.5770    5    7   27   28    0
   7 CG   C_ARO    0    0.0000   -2.7340   -1.2510   -0.4410    6    8   16    0    0
   8 CD2  C_ARO    0    0.0000   -3.5070   -1.4900    0.7130    7    9   11    0    0
   9 CE2  C_ARO    0    0.0000   -4.7820   -0.9330    0.8370    8   10   18    0    0
  10 HE2  H_ARO    0    0.0000   -5.3940   -1.1100    1.7330    9    0    0    0    0
  11 CE4  C_ALI    0    0.0000   -3.0030   -2.3360    1.8230    8   12   13   14    0
  12 HE41 H_ALI    0    0.0000   -2.1960   -1.8010    2.3820   11    0    0    0   15
  13 HE42 H_ALI    0    0.0000   -2.5840   -3.2980    1.4340   11    0    0    0   15
  14 HE43 H_ALI    0    0.0000   -3.8200   -2.5800    2.5470   11    0    0    0   15
  15 QE4  PSEUD    0    0.0000   -2.8667   -2.5597    2.1210    0    0    0    0    0
  16 CD1  C_ARO    0    0.0000   -3.2660   -0.4640   -1.4790    7   17   22    0    0
  17 CE1  C_ARO    0    0.0000   -4.5420    0.0960   -1.3550   16   18   21    0    0
  18 CZ   C_ARO    0    0.0000   -5.2920   -0.1380   -0.1990    9   17   19    0    0
  19 OH   O_HYD    0    0.0000   -6.5500    0.3880   -0.0090   18   20    0    0    0
  20 HH   H_OXY    0    0.0000   -6.8030    0.8540   -0.8190   19    0    0    0    0
  21 HE1  H_ARO    0    0.0000   -4.9580    0.7090   -2.1670   17    0    0    0    0
  22 CE3  C_ALI    0    0.0000   -2.5190   -0.2260   -2.7400   16   23   24   25    0
  23 HE31 H_ALI    0    0.0000   -3.0560    0.5020   -3.3980   22    0    0    0   26
  24 HE32 H_ALI    0    0.0000   -2.4080   -1.1840   -3.3060   22    0    0    0   26
  25 HE33 H_ALI    0    0.0000   -1.4970    0.1790   -2.5340   22    0    0    0   26
  26 QE3  PSEUD    0    0.0000   -2.3203   -0.1677   -3.0793    0    0    0    0    0
  27 HB3  H_ALI    0    0.0000   -1.4050   -2.9340   -0.2620    6    0    0    0   29
  28 HB2  H_ALI    0    0.0000   -1.0490   -1.8480   -1.6500    6    0    0    0   29
  29 QB   PSEUD    0    0.0000   -1.2270   -2.3910   -0.9560    0    0    0    0    0
  30 HA   H_ALI    0    0.0000   -0.4130   -1.5150    1.3620    5    0    0    0    0
  31 C    C_BYL    0    0.0000    1.0570   -1.7760   -0.1690    5   32   33    0    0
  32 O    O_BYL    0    0.0000    1.9871   -1.0486   -0.5134   31    0    0    0    0
  33 N    N_AMI    0    0.0000    1.1406   -3.1023   -0.1595   31    0    0    0    0
```
